# Global Linkage Map Connects Meiotic Centromere Function to Chromosome Size in Budding Yeast

**DOI:** 10.1534/g3.113.007377

**Published:** 2013-10-01

**Authors:** Anastasia Baryshnikova, Benjamin VanderSluis, Michael Costanzo, Chad L. Myers, Rita S. Cha, Brenda Andrews, Charles Boone

**Affiliations:** * Banting and Best Department of Medical Research, The Donnelly Center for Cellular and Biomolecular Research, University of Toronto, Toronto, Ontario M5S 3E1, Canada; † Department of Molecular Genetics, University of Toronto, Toronto, Ontario M5S 3E1, Canada; ‡ Department of Computer Science and Engineering, University of Minnesota, Minneapolis, Minnesota 55455; § Department of Life Sciences, Genome Damage and Stability Centre, University of Sussex, Falmer, BN1 9RQ, UK

**Keywords:** synthetic genetic array (SGA), genomics, meiosis, recombination, centromere, genetic linkage, chromosome size, double strand breaks, Rec8, Spo11, yeast, *Saccharomyces cerevisiae*

## Abstract

Synthetic genetic array (SGA) analysis automates yeast genetics, enabling high-throughput construction of ordered arrays of double mutants. Quantitative colony sizes derived from SGA analysis can be used to measure cellular fitness and score for genetic interactions, such as synthetic lethality. Here we show that SGA colony sizes also can be used to obtain global maps of meiotic recombination because recombination frequency affects double-mutant formation for gene pairs located on the same chromosome and therefore influences the size of the resultant double-mutant colony. We obtained quantitative colony size data for ~1.2 million double mutants located on the same chromosome and constructed a genome-scale genetic linkage map at ~5 kb resolution. We found that our linkage map is reproducible and consistent with previous global studies of meiotic recombination. In particular, we confirmed that the total number of crossovers per chromosome tends to follow a simple linear model that depends on chromosome size. In addition, we observed a previously unappreciated relationship between the size of linkage regions surrounding each centromere and chromosome size, suggesting that crossovers tend to occur farther away from the centromere on larger chromosomes. The pericentric regions of larger chromosomes also appeared to load larger clusters of meiotic cohesin Rec8, and acquire fewer Spo11-catalyzed DNA double-strand breaks. Given that crossovers too near or too far from centromeres are detrimental to homolog disjunction and increase the incidence of aneuploidy, our data suggest that chromosome size may have a direct role in regulating the fidelity of chromosome segregation during meiosis.

Synthetic genetic array (SGA) analysis provides an automated method for constructing yeast double mutants and mapping genetic interaction networks (Costanzo *et al.* 2010). In a typical SGA experiment, a query strain, carrying a mutation in a gene of interest, is crossed to an input array of viable deletion mutants or conditional alleles of essential genes. Sporulation and a series of selection steps produce a corresponding output array of double mutants, which can be scored for various phenotypes, including fitness, by the use of quantitative colony size measurements.

Single- and double-mutant fitness estimates can be used to identify positive and negative genetic interactions, in which the double mutant grows better or worse than expected from the combined effect of the two single mutations, respectively (Baryshnikova *et al.* 2010a). However, in addition to fitness, SGA-based colony size also reflects the efficiency at which double mutants are formed. For example, for genetically linked gene pairs, SGA analysis produces fewer double mutant cells than for gene pairs that segregate independently (Figure 1A). Consequently, double mutants involving linked gene pairs tend to form smaller colonies (Figure 1, A and B), which must be removed from genetic network analysis to prevent their misinterpretation as negative genetic interactions (Collins *et al.* 2006; Costanzo *et al.* 2010).

**Figure 1:**
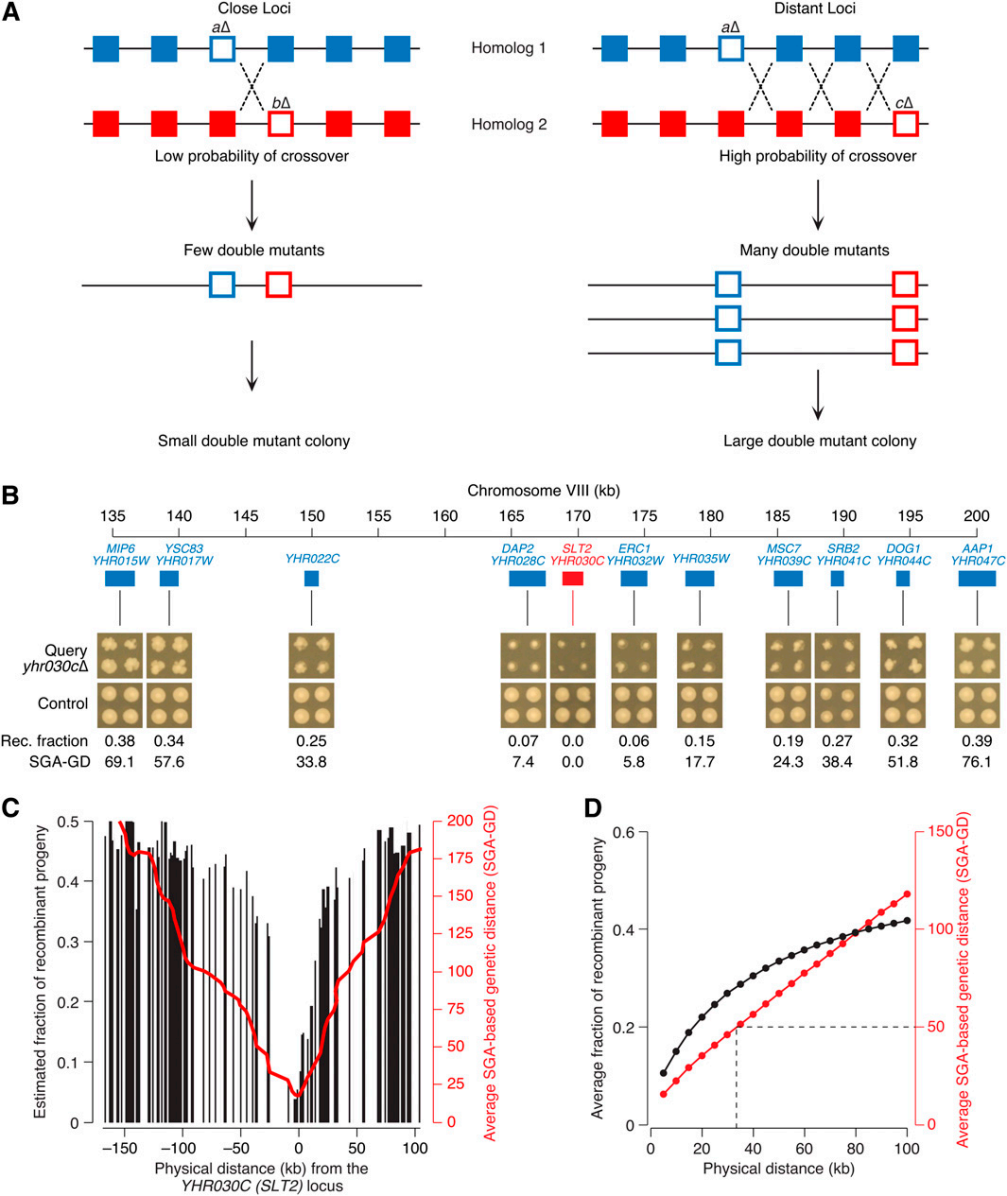
Construction of genetic linkage map based on SGA analysis. (A) In an SGA experiment, a “query” strain mutated in a gene of interest (*a*Δ) is crossed to an array of ~5000 viable deletion mutants, including those located on the same chromosome as the query (*b*Δ). Both query and array mutations (empty blue and red squares, respectively) are linked to drug-resistance cassettes, which enable selection of the double-mutant meiotic progeny, after mating and sporulation, and act as markers of meiotic recombination. Physical distance between the loci and local frequency of CO events (dotted crosses) determine the efficiency of double mutant production, which, together with double mutant fitness, defines the size of the double mutant colony formed at the final stage of SGA selection (*e.g.*, Figure 1B). Because genetic interactions are rare and are not expected to be influenced by physical distance between loci, quantitative double-mutant colony sizes can be used to estimate local recombination frequency. (B) Visual illustration of the relationship between physical distance and double-mutant colony size, which reflects recombination frequency. Images of double-mutant colonies corresponding to linked gene pairs were obtained from the SGA experiment involving the *yhr030cΔ* query strain and arranged based on their physical position on the chromosome (*yhr015wΔ–yhr047cΔ*). Double-mutant colonies of unlinked gene pairs, derived from a control experiment, are indicated for comparison. Estimated fraction of recombinant progeny (Rec. fraction; *Materials and Methods*) and SGA-GD (*Materials and Methods*) are reported for each linked double mutant. (C) A typical genetic linkage profile. Black bars correspond to the estimated frequency of recombinant progeny (*Materials and Methods*) involving the query mutation (*yhr030c*Δ) and an array mutation located on the same chromosome at the position indicated on the x-axis. The red line represents the smoothed SGA-GD between the query and the array loci (*Materials and Methods*). (D) Relationship between physical distance and recombination frequency estimates, as measured by the fraction of recombinant progeny (black line) or SGA-based genetic distance (red line). The dotted line indicates the 50 SGA-GD threshold, which corresponds to the shortest genetic distance between two unlinked loci and, on average, is equivalent to a physical distance of ~35 kb (*Materials and Methods*).

Here, we show that colony size data associated with linked gene pairs can be used to construct accurate maps of meiotic recombination throughout the yeast genome. Our analysis uncovered a previously unappreciated connection between chromosome size and centromere function during meiosis, revealing that crossovers (COs) tend to occur farther away from the centromere on larger chromosomes, which may influence chromosome segregation and aneuploidy events.

## Materials and Methods

### SGA data

SGA experiments were conducted as described in Baryshnikova *et al.* (2010a). Quantitative colony size measurements were acquired and processed using the computational pipeline described in Baryshnikova *et al.* (2010b). The Supporting Information, File S1, containing the SGA-based genetic distances (SGA-GD), is available at http://boonelab.ccbr.utoronto.ca/data/baryshnikova_2013/. A set of 16 files, one for each chromosome, contains the SGA-GD between the indicated loci. Rows and columns correspond to query and array mutants, respectively. Multiple experiments, involving the same query locus, appear as duplicated rows in each file.

### Calculating genetic distances

The recombination rate between two loci often is expressed in terms of their genetic distance, which is measured in centimorgans (cM) and refers to the average number of CO events occurring between the loci in a single meiosis (Sturtevant 1913; Griffiths *et al.* 2000). Given a population of cells undergoing meiotic division, the genetic distance between two loci can be computed by using the frequency of recombinant progeny relative to the total number of meiotic products (Griffiths *et al.* 2000). Assuming complete CO interference, a genetic distance of 50 cM would correspond to a 50% frequency of recombinants and thus would be indicative of genomic loci that segregate independently. It must be noted that the observed fraction of recombinants may differ from the actual fraction of recombinants because multiple COs occurring between two markers often remain undetected. This issue is partially addressed by the Haldane mapping function (see herein).

Using SGA data, we estimated the relative fraction of recombinant progeny for each double mutant as:

$$R=\frac{1}{2}\frac{f_{ij}}{f_{i}f_{j}}$$ (1)

where *f_i_*, *f_j_*, and *f_ij_* are the normalized colony sizes of the two single and of the double mutant, respectively, calculated as described previously (Baryshnikova *et al.* 2010b). We assume that double-mutant colony size can be used as an approximation for the number of double mutants produced by the original meiotic event and hypothesize that, in the absence of genetic linkage, just like in the absence of genetic interactions, the colony size of a double mutant, relative to wild type, should be equal to the product of the two single mutant colony sizes (*f_ij_* = *f_i_* × *f_j_*). Any difference between the observed (*f_ij_*) and the expected (*f_i_* × *f_j_*) double mutant colony size must be due to either genetic interactions or genetic linkage. Because genetic interactions are rare and do not normally depend on physical distance between loci, the average colony size of double mutants involving genes located in close proximity to one another should only reflect genetic linkage, whereas the contribution of genetic interactions can be considered negligible (Figure 1C). A similar reasoning pertains to potential differences in sporulation efficiency, which might also affect double-mutant colony size. In addition to being rare and independent from physical positioning, sporulation defects are unlikely to be manifested in heterozygous diploids from which double mutants are produced in SGA. The ratio of observed and expected double-mutant fitness is multiplied by a factor of 0.5, such that R = 0.5 for genetically independent loci (*f_ij_* = *f_i_* × *f_j_*).

Recombinant fraction R counts the number of recombinant spores relative to all spores generated by meiosis and can be used as a measure of recombination among genomic loci. However, R often underestimates the true recombination rate because it does not account for recombination events that do not produce double mutants. For example, multiple CO events produce a parental arrangement of alleles and the resulting meiotic products do not contribute to recombinant frequency.

Several normalization methods have been proposed to correct for this inaccuracy (Morton 2004). One of the simplest methods, known as Haldane’s mapping function (Hartl and Jones 2009), is based on the assumption that recombination events are distributed randomly within a given region and, thus, the probability of observing *i* recombination events follows the Poisson distribution:

$$f\left(i\right)=\frac{e^{-m}m^{i}}{i!}$$ (2)

where *m* is the mean number of recombination events in the region per meiosis. Haldane’s mapping function assumes that multiple CO events occur independently from each other and do not experience interference (Hartl and Jones 2009). Although this is a fairly strong assumption, CO interference is unlikely to have a strong impact on our estimates of recombination frequency based on colony sizes. As a result, we believe that the adoption of the Haldane’s mapping function is justified.

Recombinants comprise 50% of the spores derived from meioses in which at least one recombination event occurred in the region. Thus, recombinant fraction R can be expressed as:

$$R=\frac{1}{2}\left(1-e^{-m}\right)$$ (3)

where e^–^*^m^* is the number of meioses with zero recombination events:

$$f\left(0\right)=\frac{e^{-m}m^{0}}{0!}=e^{-m}$$ (4)

From Equation 4, we can derive *m*, which would equal to the probability of observing a recombination event in a given region:

m=−ln(1−2R) (5)

Recombination rate between loci is often expressed in terms of their genetic distance, measured in Morgans, such that 1 Morgan equals 1 recombinant product per meiosis. Because each recombination event produces two recombinant products, genetic distance *M* is derived from Equation 5 as:

$$M=\frac{1}{2}m=-\frac{1}{2}\ln\left(1-2R\right)$$ (6)

1 centimorgan (cM) equals 0.01 Morgans.

Compared with classical genetic studies based on small scale tetrad analyses (Cherry *et al.* 2012), genetic distances derived from SGA double-mutant colony sizes seem to systematically overestimate recombination rates. To emphasize this quantitative difference and prevent confusion, we avoid the term “centimorgan” and describe our data in terms of SGA-GD. A unit of SGA-GD is equivalent to a centimorgan in the context of SGA data and a genetic distance of 50 SGA-GD corresponds to a recombinant frequency of 50% and thus is indicative of genomic loci that segregate independently (Griffiths *et al.* 2000) (Figure 1, C and D).

### Constructing genetic linkage maps

For each SGA experiment, we calculated the genetic distances between the query locus and all array loci located on the same chromosome. Because even inviable double mutants or double mutant failing to germinate form small residual colonies, raw genetic distances were normalized such that the genetic distance between the gene and itself is zero.

Each chromosome was split into a set of consecutive nonoverlapping bins of 5 kb each. Query and array genes were assigned to bins based on their chromosomal position, as reported by the *Saccharomyces* Genome Database (SGD, www.yeastgenome.org; accessed in February 2011). A genetic linkage map G for chromosome C was generated as a n × n matrix, where n is the total number of bins in C, and the value G*_ij_* corresponds to the average of SGA-based genetic distances for query-array gene pairs where queries belong to bin *i* and arrays belong to bin *j*. The consolidated genetic linkage map G′ (Figure S1) was calculated by averaging query-array and array-query genetic linkage maps for the same positions:

$$G^{\prime }=\frac{G+G^{T}}{2}$$ (7)

Averaging of query and array genetic linkage maps was applied to all chromosomes, with the exception of chromosome III, because of its peculiar role in the SGA selection process (Figure S3). In addition, the left arms of chromosomes V and XIV, harbor two of the markers used during the SGA double mutant selection: *YEL063C* (*CAN1*) and *YNL268W* (*LYP1*). As a result, all SGA screens show linkage in the corresponding areas of chromosomes V and XIV and gene-specific linkages cannot be reported (gray areas in Figure S1, panels V and XIV).

### Hotspot analysis

We estimated the recombination frequency at each position x along the chromosome by computing the average SGA-based genetic distance within the interval [x – 2, x + 2], corresponding to a 25-kb interval centered on position x (5 bins × 5 kb/bin). The resulting profile of recombination frequency was smoothed using a moving average filter over five consecutive positions.

We identified 347 putative recombination hotspots defined as local maxima of recombination frequency, measured in SGA-GD per kilobase, that were greater than the average genome recombination frequency. The distribution of these peaks coincided well with known genomic loci associated with high levels of meiotic recombination (Lichten and Goldman 1995). For example, we identified 8 of 9 most well-characterized recombination hotspots (*HIS2*, *HIS4*, *ARG4*, *CYS3*, *DED81*, *ARE1-IMG1*, *CDC19*, and *THR4*; data not shown).

Meiotic recombination in yeast has been investigated on a genome-wide scale with the use of a variety of experimental approaches (Baudat and Nicolas 1997; Borde *et al.* 2004; Blitzblau *et al.* 2007; Buhler *et al.* 2007; Mancera *et al.* 2008; Pan *et al.* 2011). These studies focused primarily on mapping the initial phases of recombination, including Spo11 DNA binding (Gerton *et al.* 2000; Pan *et al.* 2011) and DSB formation (Baudat and Nicolas 1997), as well as early recombination intermediates, such as single-stranded DNA produced by resection (Blitzblau *et al.* 2007; Buhler *et al.* 2007). In addition, CO and non-CO recombination outcomes have been identified by genotyping single-nucleotide polymorphisms in parents and progeny of sampled meioses (Mancera *et al.* 2008).

We compared the recombination profiles obtained from these published studies of meiotic recombination to those derived from our SGA-based genetic linkage analysis. The physical locations of recombination hotspots were downloaded from the supplementary material of each publication, as described in Table S1.

Our data showed good precision and sensitivity in detecting recombination hotspots reported in other studies (Figure S4). For example, 65% and 77% of hotspots reported by Pan *et al.* (2011) and Mancera *et al.* (2008), respectively, are located within 10 kb from a hotspot identified by our SGA-based dataset (Figure S4).

### Relationship between chromosome size and centromere linkage

#### SGA data:

All gene pairs located on the same chromosome were divided into *cis*-pairs (located on the same side of the centromere) and *trans*-pairs (located on opposite sides on the centromere). Gene pairs in each group were then distributed into discrete 5-kb bins according to their physical distance. Within each bin, genetic distances were averaged. The extent of centromere linkage was computed as the physical distance of smallest (closest to the centromere) bin of *trans*-pairs where the genetic distance between loci was on average equal or greater than 50 SGA-GD.

#### Other studies of meiotic recombination:

Data relative to the location of recombination hotspots were downloaded from the supplementary websites of the respective publications, as indicated in Table S1. Linkage around the centromere was defined as the shortest physical distance between two hotspots located on opposite sides of the centromere. As a control for centromere-unrelated linkage, we calculated the average physical distance between any pair of consecutive hotspots.

In addition, a genetic map was downloaded from the *Saccharomyces* Genome Database (http://downloads.yeastgenome.org/chromosomal_feature/genetic_map.tab). Linkage around the centromere was defined as the shortest physical distance between two genetically independent loci (50 cM) located on opposite sides of the centromere. As a control for centromere-unrelated linkage, we calculated the expected physical distance between two unlinked loci by using the average number of centimorgans per kilobase reported for each chromosome at http://www.yeastgenome.org/pgMaps/pgMap.shtml.

We computed Pearson correlation coefficients and the associated significance *p*-values between chromosome size and the estimated extent of centromere-related and centromere-unrelated linkage (Figure S5). Given that a number of published studies reported an overall increase in meiotic recombination rates for the four smallest chromosomes (Kaback *et al.* 1992; Gerton *et al.* 2000; Blitzblau *et al.* 2007; Cherry *et al.* 2012), we also computed correlations between chromosome size and linkage extent following the removal of chromosomes I, III, VI, and IX. We found that any correlation observed between chromosome size and centromere-unrelated linkage, was completely abolished when the 4 smallest chromosomes were excluded from the analysis (Figure S5). In contrast, centromere-related linkage was generally unaffected (Figure S5).

### Measuring centromere-bound cohesin clusters

We obtained quantitative profiles of meiotic cohesin binding from two recent genome-wide studies (Glynn *et al.* 2004; Kiburz *et al.* 2005), as well as a third study in which the authors measured cohesin binding along four chromosomes at different time points (Kugou *et al.* 2009) (Table S1). Profiles were smoothed using a moving average filter applied across 25 consecutive positions. The extent of centromere-bound cohesin clustering was measured as the width of the centromere-centered peak of cohesin binding. Peak width was defined as the distance between the two farthest chromosomal positions, on either side of the centromere, where cohesin binding was greater than global genomic average (a simple *b* > *GGA* cutoff was applied, where *b* is cohesin binding and *GGA* is global genomic average).

## Results

### Construction of genetic linkage map based on SGA analysis

In SGA analysis, double-mutant colony size reflects both the efficiency of double mutant formation (*i.e.*, the fraction of double mutants among the meiotic progeny of a heterozygous diploid) and double-mutant fitness (*i.e.*, growth rate). While double-mutant fitness defects generally are rare and independent from chromosomal position (Costanzo *et al.* 2010), a reduced efficiency of double mutant formation is often observed for gene pairs located closely on the same chromosome (Figure 1, A and B). For linked gene pairs, formation of double mutants depends on meiotic recombination, whose frequency increases with physical distance at a rate that is specific to a given genomic region. Taking advantage of our growing genome-wide SGA dataset, we examined the potential to estimate region-specific recombination rates from the colony sizes of the double mutants carrying genes located close to one another on the same chromosome (Figure 1 and the section *Materials and Methods*).

From a collective set of ~3800 genome-wide SGA experiments, we obtained quantitative double mutant colony sizes for ~1.2 million gene pairs co-localized on the same chromosome (File S1). Colony sizes were measured in pixel units from digital images of double-mutant plates (Figure 1B) and normalized for experimental systematic effects with the use of a computational protocol developed for genetic interaction analysis (Baryshnikova *et al.* 2010b). For each gene pair, we used single and double mutant−normalized colony sizes to estimate the relative frequency of recombination progeny for each double mutant (Figure 1C and the section *Materials and Methods*). Using this frequency, we calculated the genetic distance between the corresponding loci, which is referred to as SGA-based genetic distance, or SGA-GD. A unit of SGA-GD is comparable with a centimorgan, a widely used measure of genetic linkage and recombination frequency. A genetic distance of 50 SGA-GD corresponds to a recombinant frequency of 50% and thus is indicative of genomic loci that segregate independently (Griffiths *et al.* 2000) (Figure 1C and the section *Materials and Methods*).

We found that SGA-based genetic distance correlated linearly with physical distance, a trend that is expected if double-mutant colony size truly reflects recombination frequency (Figure 1D). On average, genes located more than 35 kb apart tended to segregate independently, as their genetic distance was 50 SGA-GD or greater (Figure 1D). In contrast, early genetic tests, based on classical tetrad analysis, estimated that unlinked loci are normally positioned at ~139 kb (Cherry *et al.* 2012), a distance ~4-fold greater than our estimate of 35 kb. This difference suggests that SGA data overestimate the frequency of meiotic recombination. One potential explanation for this result is that, in SGA, double-mutant colony sizes are quantified 30−40 generations after the original meiotic event. At that stage, many colonies have reached saturation and small growth disadvantages, experienced by weakly linked gene pairs, might be diminished, thus causing an overestimation of local recombination rates. Despite this limitation, every chromosomal region in our dataset should be equally affected by this phenomenon, which therefore is expected to have a minor impact on the results of our comparative analyses.

**Figure 2:**
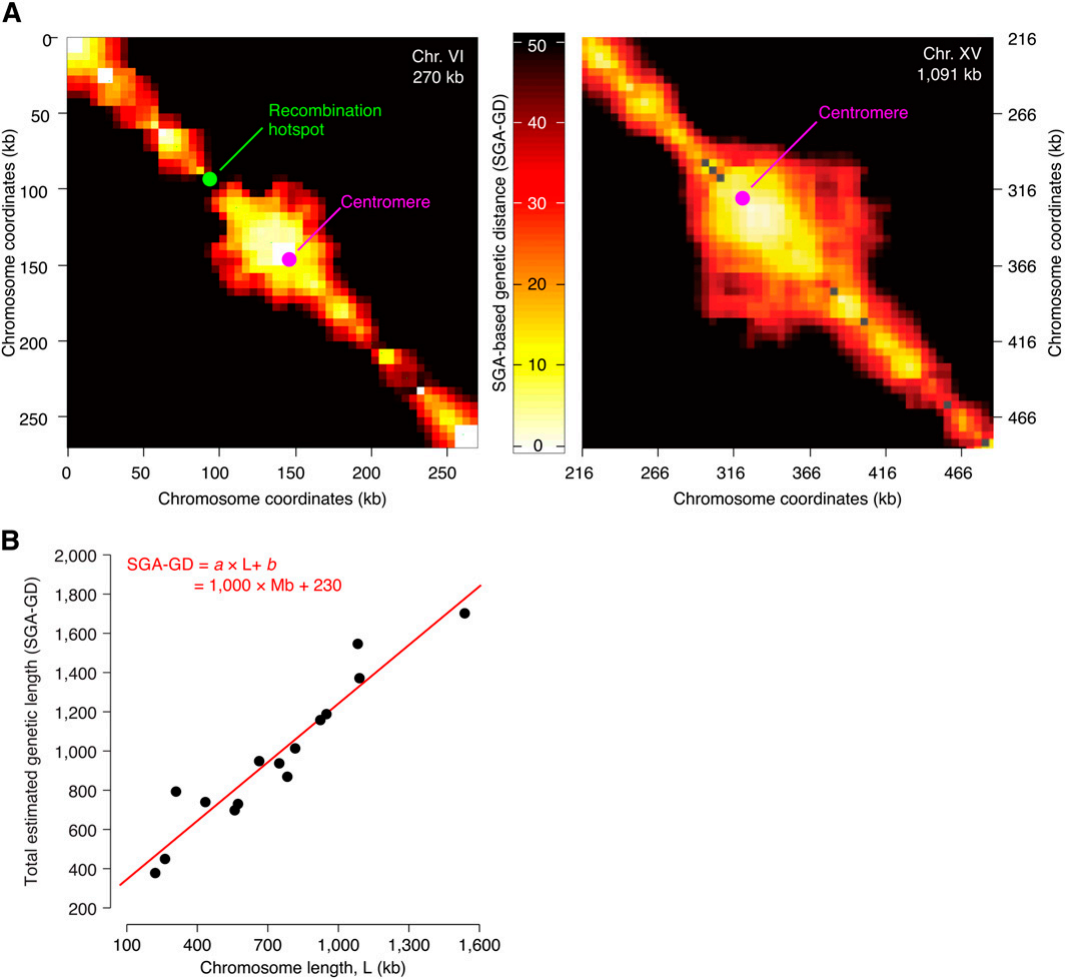
Pericentric linkage and chromosome size dependent recombination rates in linkage map based on SGA analysis. (A) The genetic linkage map of chromosome VI and part of chromosome XV. The horizontal and the vertical axes represent chromosomal coordinates; a third dimension indicating the SGA-GD between the corresponding chromosomal regions is represented by the intensity of color of each point in the image (*Materials and Methods*). The two chromosomes are drawn to scale. The green dot labeled as “Recombination hotpot” indicates the position of the most active DSB hotspot on chromosome VI, as reported previously (Pan *et al.* 2011). (B) Relationship between total physical and total genetic length of yeast chromosomes. Each data point corresponds to a chromosome. The total estimated genetic length of a chromosome (SGA-GD) was calculated from the physical length (kilobases) and the average SGA-GD/kb ratio of each chromosome. The definition of SGA-GD is described in the section *Materials and Methods*. The red line and the associated equation correspond to a linear fit to the data.

We used the set of SGA-based genetic distances between all tested gene pairs to construct a genome-wide, ~5 kb-resolution, genetic linkage map (*Materials and Methods*). This map revealed large blocks of linkage, where recombination is rare and neighboring loci are inherited jointly (Figure 2A; Figure S1). Linkage blocks were separated by relatively shorter stretches of loci that appeared to be genetically unlinked and thus likely to harbor recombination hotspots (Figure 2A; Figure S1; and *Materials and Methods*). Our genetic linkage data were reproducible (Figure S2 and Figure S3) and consistent with previous studies of meiotic recombination (Figure S4; and the section *Materials and Methods*). In particular, we confirmed the relationship, previously observed in yeast and in other organisms, between recombination events and chromosome size whereby the total number of COs per chromosome follows a simple linear model *a*L + *b*, where L is chromosome size, while *a* and *b* are constants (Figure 2B) (Riles *et al.* 1993; Kaback 1996; Stahl *et al.* 2004; Mancera *et al.* 2008; Fledel-Alon *et al.* 2009).

### Positive correlation between chromosome size and the size of pericentric linkage regions

**Figure 3:**
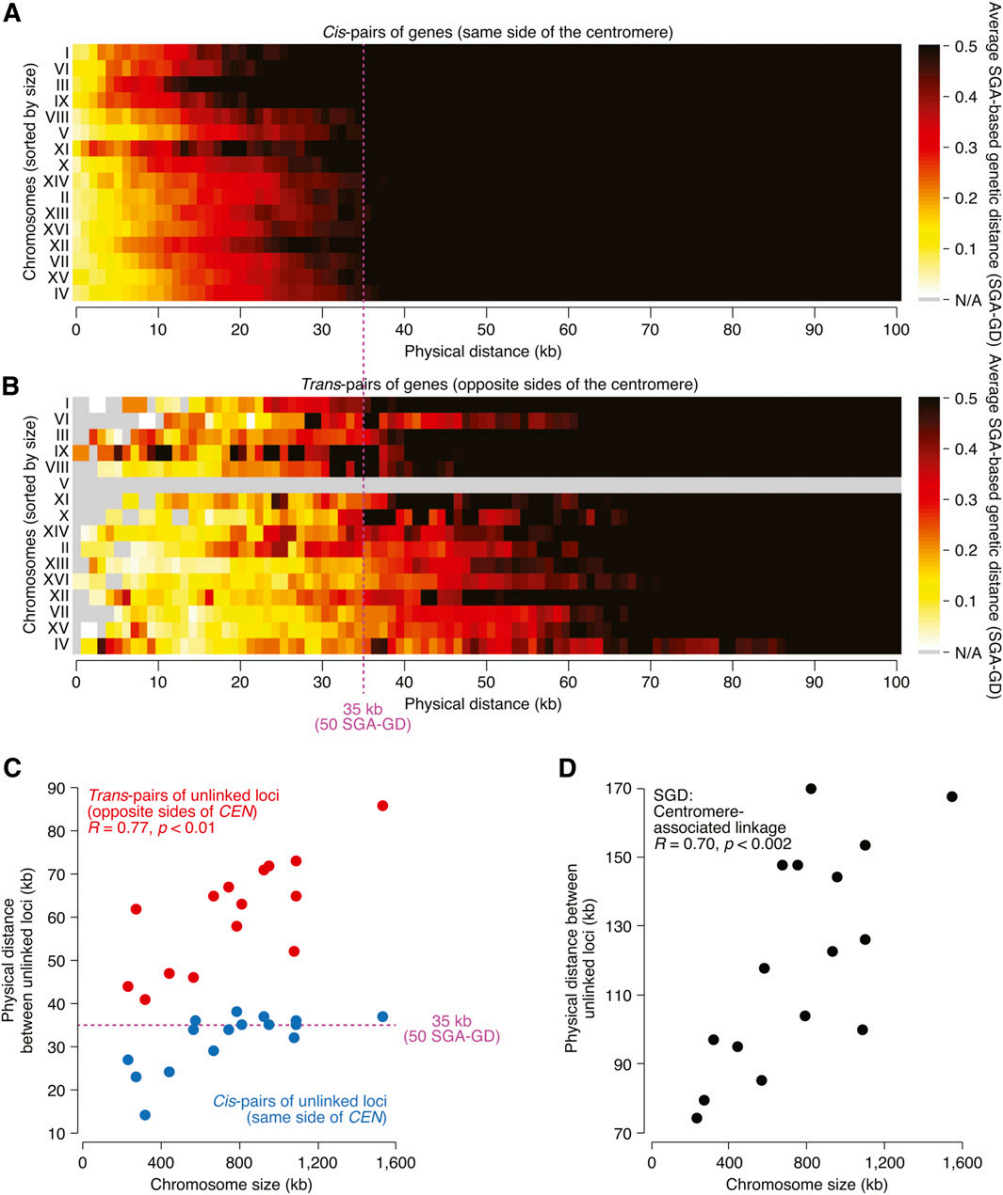
Relationship between chromosome size and meiotic recombination around centromeres. (A–B) Distribution of estimated genetic distances for gene pairs located at a range of physical distances. Gene pairs located on the same chromosome (ordered by chromosome size and listed on the y-axis) were grouped according to their physical distance (x-axis). Within each group, genetic distances were averaged and visualized as a heatmap, where brighter colors correspond to shorter genetic distances and stronger linkage. Gene pairs located on the same side of the centromere (*cis*; **A**) or on opposite sides of the centromere (*trans*; B) were analyzed separately. Data for gene pairs spanning the centromere on chromosome V were not available due to the experimental constraints of the SGA method (*Materials and Methods*). The dotted line indicates the average genetic distance (35 kb) of unlinked loci (50 SGA-GD) (Figure 1D and the section *Materials and Methods*). (C) Relationship between chromosome size and the shortest physical distance between two unlinked loci, positioned on the same (*cis*) or on opposite sides (*trans*) of the centromere. Each data point corresponds to a chromosome. Data for *trans*-centromere gene pairs on chromosome V were not available due to the experimental constraints of the SGA method (*Materials and Methods*). *R* corresponds to Pearson correlation coefficient. (D) The shortest physical distance between two unlinked loci, positioned on opposite sides of the centromere, was calculated for each chromosome using the genetic map downloaded from the *Saccharomyces* Genome Database (Cherry *et al.* 2012), and plotted against chromosome length. *R* corresponds to Pearson correlation coefficient.

In our genetic linkage map, the centromeres of all chromosomes were associated with extensive regions of genetic linkage (Figure 2A; Figure S1), consistent with centromeric suppression of meiotic recombination (Brar and Amon 2008; Chen *et al.* 2008; Mancera *et al.* 2008; Pan *et al.* 2011). We examined pericentric linkage for each chromosome by measuring the shortest physical distance between two unlinked loci positioned on the same side (*cis*) or on opposite sides (*trans*) of the centromere (Figure 3, A−C, and the section *Materials and Methods*). The shortest distance between *cis*-pairs of unlinked loci generally was constant for all chromosomes and matched the global genomic average of 35 kb, with the exception of the four smallest chromosomes (I, III, VI, and IX), whose average recombination rate is greater, as documented previously (Kaback *et al.* 1992, 1999; Pan *et al.* 2011; Cherry *et al.* 2012) and consistently with the *a*L + *b* model. In contrast, the shortest distance between *trans*-centromeric pairs of unlinked loci was notably greater than 35 kb for every chromosome (Figure 3, B and C), consistent with pericentric suppression of meiotic recombination (Brar and Amon 2008; Chen *et al.* 2008; Mancera *et al.* 2008; Pan *et al.* 2011). Furthermore, the shortest distance between *trans*-centromeric pairs varied from chromosome to chromosome and correlated significantly with chromosome size (*R* = 0.77, *p* < 0.01; Figure 3C). This correlation indicates that, on larger chromosomes, COs tend to occur farther away from the centromere, resulting in the formation of larger linkage regions. We confirmed the relationship between chromosome size and pericentric linkage using several published datasets (Figure S5), including large-scale mapping of individual CO events (Mancera *et al.* 2008) and the classical yeast genetic map compiled from traditional tetrad analysis (Cherry *et al.* 2012) (Figure 3D).

### Negative correlation between chromosome size and levels of pericentric DSBs

**Figure 4:**
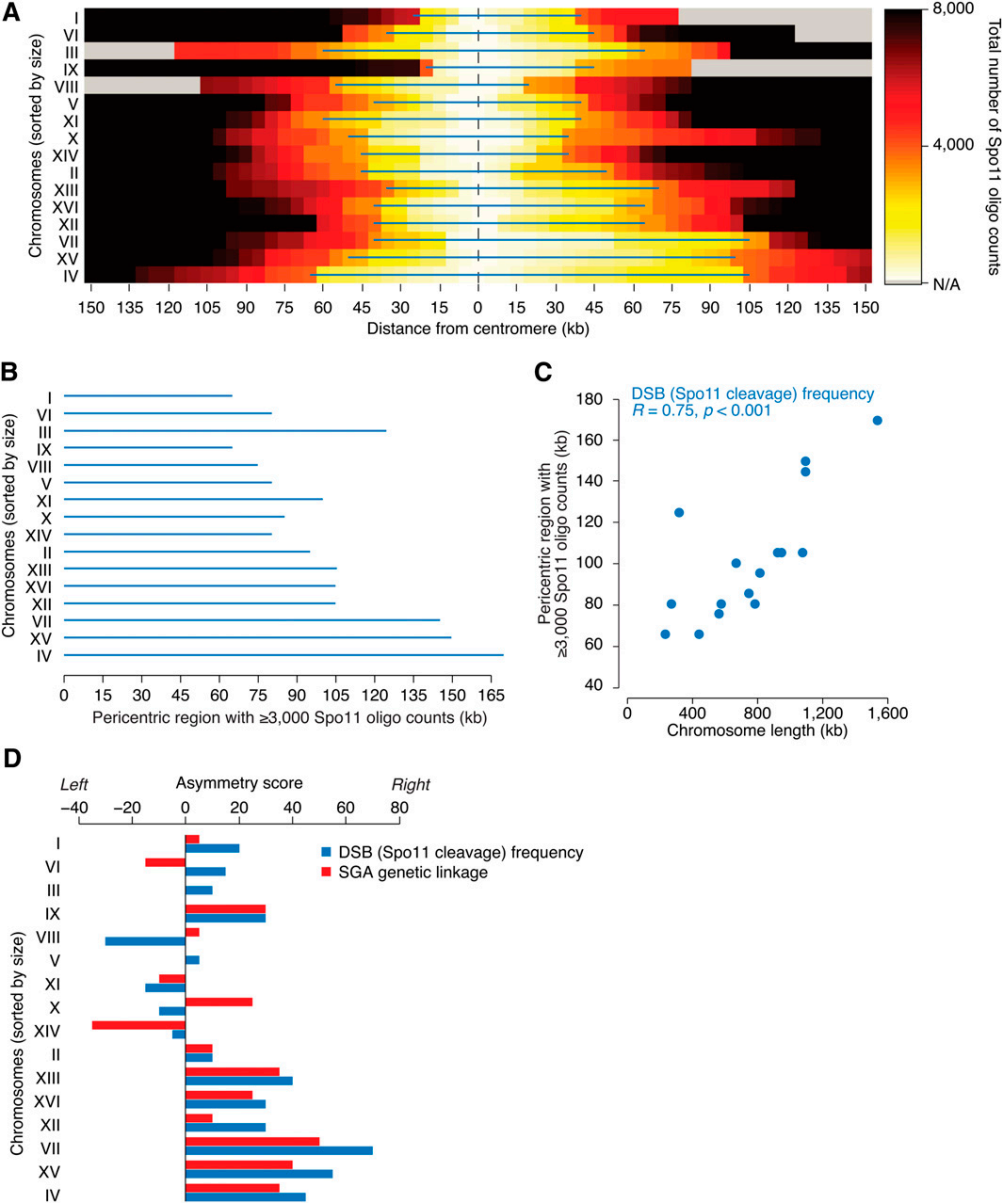
Relationship between chromosome size and frequency of DSB formation within pericentric regions. (A) The number of Spo11-bound oligos, a readout for meiotic DSBs, around centromeres was calculated from data reported in (Pan *et al.* 2011). The color scale represents the number of Spo11-bound oligos recovered between the centromere and loci within the physical distance indicated on the x-axis. The blue lines mark the DSB-repressed regions around the centromeres where less than 3000 Spo11-bound oligos were recovered. (B) Size of the DSB-repressed regions around the centromeres in A, represented in order of chromosome size. (C) Relationship between chromosome size and the size of pericentric DSB-repressed regions in panel A. *R* corresponds to Pearson correlation coefficient. Similar results were obtained with variable thresholds for the number of Spo11-bound oligos (data not shown). (D) Asymmetry of pericentric distributions of DSBs and meiotic recombination. An asymmetry score for pericentric recombination was calculated as the difference between the left and the right pericentric regions with at least 3000 Spo11 oligos detected [DSB (Spo11 cleavage) frequency] or presenting a genetic distance greater or equal to 50 SGA-GD from the centromere (SGA genetic linkage). Asymmetry scores, expressed in kilobases, are plotted for all chromosomes, sorted by their size.

Meiotic recombination begins with programmed catalysis of DNA double-strand breaks (DSBs) by Spo11, an evolutionarily conserved type II topoisomerase-like endonuclease (Bergerat *et al.* 1997; Keeney *et al.* 1997). Genome-wide distributions of DSBs and COs exhibit a strong correlation (Figure S4) (Chen *et al.* 2008; Mancera *et al.* 2008; Pan *et al.* 2011). Moreover, similarly to COs, DSBs are repressed near centromeres (Gerton *et al.* 2000; Buhler *et al.* 2007; Pan *et al.* 2011). To test whether the DSB repression might also be related to chromosome size, we examined the genome-wide DSB map based on high-throughput sequencing of short Spo11-bound oligonucleotides (Spo11-oligos), a byproduct of an early step in meiotic DSB repair and an established readout for the occurrence of a DSB (Pan *et al.* 2011). We found that, similarly to the suppression of recombination frequency, the extent of DSB suppression around centromeres appears to be directly proportional to chromosome size (Figure 4). For instance, in order to observe the same number of Spo11-oligos between two *trans*-loci spanning the centromere, their physical distance must be greater on larger chromosomes compared to smaller ones (*R* = 0.75; *p* < 0.001; Figure 4, B and C).

The aforementioned analyses suggest that chromosome size may regulate both frequencies of meiotic DSBs and COs around centromeres. Nevertheless, we observed some exceptional cases where a previously mapped DSB hotspot occurred within a pericentric linkage region (Figure S1), consistent with the fact that not all meiotic DSBs are repaired via the CO-generating inter-homolog repair pathway (Schwacha and Kleckner 1994). We also found that pericentric linkage regions were often asymmetrical with respect to DSB frequency, as well as linkage intensity (Figure 2A; Figure S1; Figure 4D), suggesting that chromosome size and distance from the centromere are not the only factors determining the frequency of meiotic DSB catalysis and/or the manner in which these breaks are repaired.

### Positive correlation between chromosome size and pericentric association of meiotic cohesin subunit Rec8

**Figure 5:**
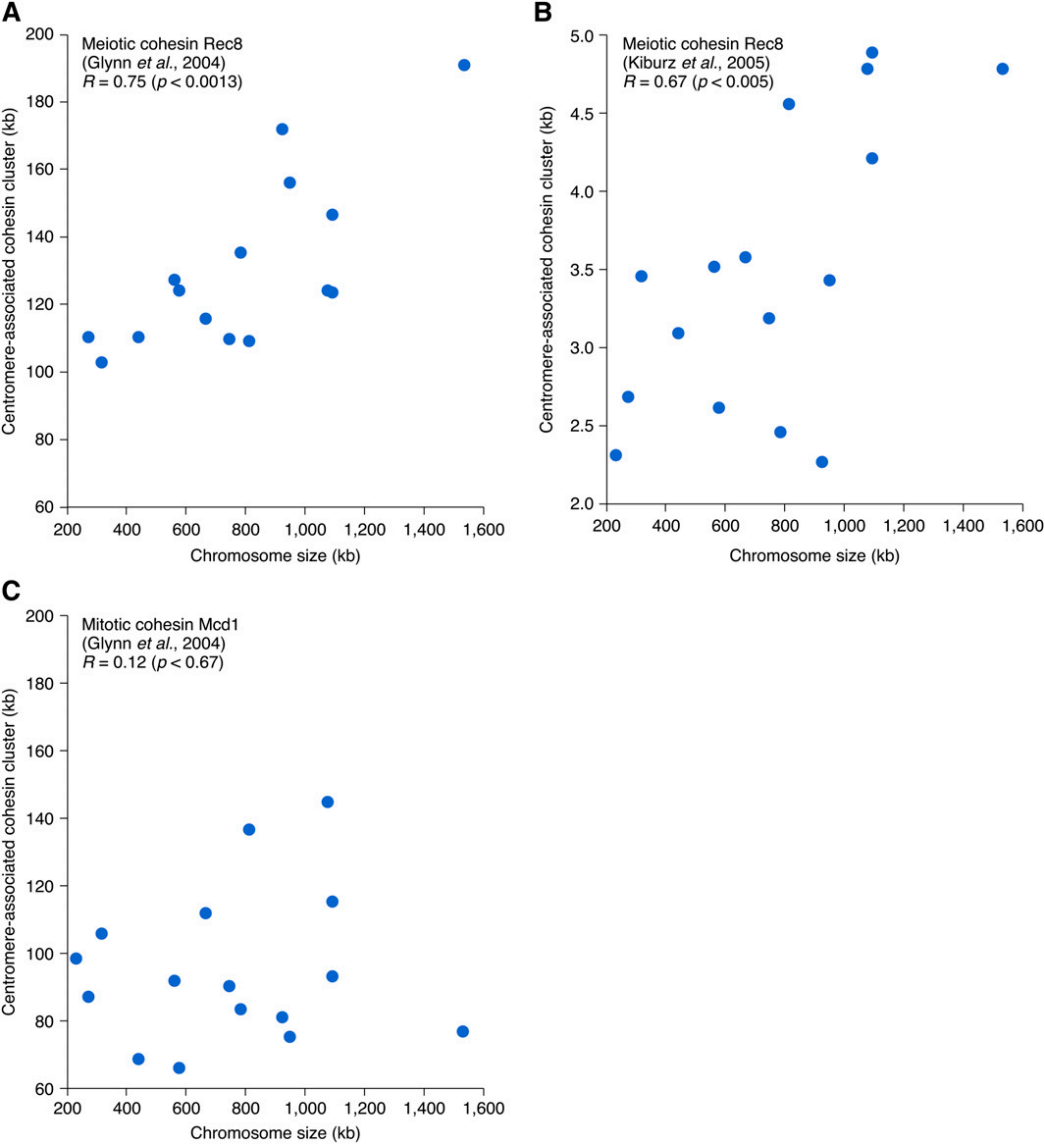
Relationship between chromosome size and the extent of the centromere-associated clustering of Rec8 and Mcd1/Scc1. (A, B) Positive correlation between chromosome size and the meiotic cohesin component Rec8. Quantitative profiles of Rec8 binding at 4 hr (A) and 5 hr (B) after the initiation of meiosis, corresponding to meiotic metaphase/anaphase, were obtained from Glynn *et al.* (2004) and Kiburz *et al.* (2005), respectively (*Materials and Methods*). Each data point corresponds to a chromosome. *R* corresponds to Pearson correlation coefficient. The correlation is apparent in the two independent studies despite the noticeable differences in the sizes of the centromere clusters of Rec8, likely reflecting differences in data collection and/or analyses. (C) Relationship between chromosome size and the extent of centromere-associated clustering of the mitotic cohesin component Mcd1/Scc1 in metaphase arrested cells (Glynn *et al.* 2004).

Chromatin structure regulates both the formation and repair of meiotic DSBs. Of particular interest in the current context is the status of sister chromatid cohesion, which impacts both processes. Specifically, occurrence of meiotic DSBs is confined within the regions that are devoid of cohesin molecules, referred to as chromatin loops (Blat and Kleckner 1999). Sister chromatid cohesion also antagonizes COs, generating interhomolog DSB repair by favoring an alternative intersister repair pathway (*e.g.*, Niu *et al.* 2005). Combining these considerations with the fact that cohesin molecules are enriched at centromeres (Glynn *et al.* 2004; Kiburz *et al.* 2005) raised the possibility that the effect of chromosome size might be linked to cohesin association at centromeres. To explore this possibility, we examined the relationship between chromosome size and the extent of centromere-associated clustering of Rec8, an evolutionary conserved meiotic subunit of the multiprotein cohesin complex. We found that the width of Rec8 centromere-binding peak, measured from two different genome-wide surveys of Rec8 distribution during meiotic metaphase/anaphase (Glynn *et al.* 2004; Kiburz *et al.* 2005), correlates with chromosome size (*R* = 0.75 and *R* = 0.67, respectively, Figure 5, A and B and the section *Materials and Methods*). In contrast, for mitotic cohesion, the centromere association of Mcd1/Scc1 does not show size dependent variation (Figure 5C), suggesting that the size dependence might be specific to meiotic centromere function.

## Discussion

Meiosis is a specialized cell division program, during which a single round of genome replication is followed by two successive rounds of genome segregation. During the first meiotic division (meiosis I) sister chromatids remain associated with one another, whereas homologs segregate to opposite poles. The accuracy of this process depends on the persistent centromeric cohesion and the formation of interhomolog COs, which facilitate homolog alignment, orientation and bipolar attachment to the meiotic spindle (Bishop and Zickler 2004; Pinsky and Biggins 2005).

Frequency and positioning of COs impact chromosome segregation because COs that are too distal from centromeres are not as effective in facilitating bipolar attachment (Koehler *et al.* 1996; Ross *et al.* 1996; Lacefield and Murray 2007), whereas COs that are too close to centromeres can lead to precocious separation of sister chromatids (Rockmill *et al.* 2006). Indeed, centromere proximal CO formation is reduced in many organisms (Brar and Amon 2008) and may represent a general mode of regulating the fidelity of meiotic chromosome segregation (Rockmill *et al.* 2006; Chen *et al.* 2008). In humans, failure to form COs or their abnormal distribution cause aneuploidies and severe congenital conditions such as Down syndrome (Hassold *et al.* 2007).

Here, we show that colony size data associated with linked gene pairs can be used to build accurate maps of meiotic recombination throughout the yeast genome. Our study differs from previous genomic analysis of meiotic recombination in that we obtained our data from an isogenic strain, rather than a hybrid carrying alleles of two divergent genetic backgrounds (Chen *et al.* 2008; Mancera *et al.* 2008). Our results confirm previous findings that: (1) the total number of CO events per chromosome increases with chromosome size, (2) the number of COs per unit length is greater in shorter chromosomes than longer ones, and (3) meiotic recombination occurs at lower frequencies in the vicinity of centromeres. We also present a previously unappreciated relationship between chromosome size and meiotic recombination, where chromosome size shows a negative correlation with pericentric CO density and DSB frequency, but a positive correlation with Rec8 association.

Spo11-mediated catalysis of DSBs initiates meiotic recombination. Observations that sister chromatid cohesion antagonizes CO formation by down-regulating both DSB catalysis and CO-generating inter-homolog DSB repair (*e.g.*, Niu *et al.* 2005; Pan *et al.* 2011) suggest that the effect of chromosome size on pericentric recombination might be mediated by size-dependent loading of Rec8. Notably, however, because the Rec8 analysis was performed on cells in meiosis I (*i.e.*, after completion of meiotic recombination) (Glynn *et al.* 2004; Kiburz *et al.* 2005), it is possible that the observed association between chromosome size and centromeric enrichment of Rec8 is a consequence rather than the cause. For example, in mitosis, the extent of pericentric cohesin association is negatively correlated with tension between sister kinetochores (Eckert *et al.* 2007), possibly because mechanical tension acts toward displacing cohesin from pericentric regions. If a similar feedback system functions in meiosis, then perhaps the larger cohesin occupancy at the centromeres of larger chromosomes after meiotic recombination reflects the fact that their kinetochores experience reduced spindle tension, which may follow from more distal COs.

The distribution of CO events per yeast chromosome can be described by the *a*L + *b* model (Riles *et al.* 1993; Stahl *et al.* 2004; Mancera *et al.* 2008). This model is consistent with the hypothesis that yeast chromosomes undergo two different types of CO events: one type occurs at a constant rate per kb (parameter *a*), causing the total number of CO events to depend directly on chromosome size; the other CO type occurs at a constant rate per chromosome (parameter *b*) and thus is chromosome-size independent. Although the relative contribution of each type of CO events is still unclear, this model implies that, because of their small size, the shortest chromosomes would be relatively enriched for the *b*-type CO events and present a greater total recombination rate per kilobase, a trend that was indeed observed by earlier studies (Kaback *et al.* 1992; Riles *et al.* 1993; Kaback *et al.* 1999; Stahl *et al.* 2004; Mancera *et al.* 2008; Pan *et al.* 2011; Cherry *et al.* 2012). It would be reasonable to speculate that the chromosome size-independent *b*-type CO may correspond to the so-called “obligate” CO (Jones 1987), which ensures that even the smallest possible chromosome, which would consist of just a centromere and two telomeres, undergoes at least one physical exchange with its homolog. This obligate physical exchange would be required to tether homologs together and facilitate their bi-orientation with respect to the spindle, thus ensuring proper chromosome alignment and segregation (Bishop and Zickler 2004; Pinsky and Biggins 2005). Because segregation is impaired when COs occur too close or too far from the centromere (Koehler *et al.* 1996; Ross *et al.* 1996; Rockmill *et al.* 2006; Lacefield and Murray 2007), the centromere may play a role in determining the positioning of the obligate CO. This centromere function may be particularly relevant in humans where unregulated recombination near centromeres leads to aneuploidy and severe developmental abnormalities, including Down syndrome (Hassold *et al.* 2007). Importantly, a recent analysis of the human genome-wide recombination map showed that, similarly to yeast, the total number of CO events per chromosome follows the *a*L + *b* model, where *b* ≈ 1 (Fledel-Alon *et al.* 2009).

The relationship between chromosome size and the extent of pericentric linkage and Rec8 enrichment is likely to be linked to an essential requirement that centromeric cohesion remains intact during the first meiosis (Brar and Amon 2008). Indeed, budding yeast *zip1* mutants that are impaired in centromeric cohesin loading display promiscuous recombination in the vicinity of their centromeres (Chen *et al.* 2008; Bardhan *et al.* 2010), suggesting a mechanistic link between meiotic centromere function and suppression of pericentric recombination. Here, we provide further evidence for such a link. In addition, we show that chromosome size itself might regulate both pericentric recombination and meiotic centromere function.

Interestingly, in yeast *Saccharomyces cerevisiae* the inactivation of the spindle checkpoint causes larger chromosomes to missegregate more often than smaller chromosomes (Shonn *et al.* 2000; Lacefield and Murray 2007). Because an essential function of the spindle checkpoint is to monitor bipolar attachment of chromosomes (Pinsky and Biggins 2005), this finding suggests that chromosome size may impact the efficacy of homolog biorientation. For example, COs may tend to occur further away from the centromere of larger chromosomes leading to a defect in spindle attachment (Shonn *et al.* 2000; Lacefield and Murray 2007). This observation is particularly intriguing because all budding yeast centromeres appear to have the same point structure (Brar and Amon 2008).

There is a precedent for size-dependent loading of chromosomal-bound proteins: the extent of Smc5/Smc6 loading during mitotic S-phase is chromosome size dependent, and is proposed to be controlled by a chromosome structure-based mechanism (Kegel *et al.* 2011). It is possible that the structural properties of larger chromosomes may differ from those of smaller chromosomes and may control Rec8 loading at centromeres. Indeed, analysis of early Rec8 loading at the onset of meiotic S-phase suggests a positive correlation between chromosome size and the initial pericentric clustering of Rec8 (Figure S6). Regardless of the mechanism, our data points to the evolution of a balanced system, where the need to exclude COs from centromeres to ensure sister centromere cohesion and homolog centromere pairing creates a tendency for incorrect kinetochore-spindle attachment that is subsequently corrected by the spindle checkpoint (Shonn *et al.* 2000; Lacefield and Murray 2007).

## Supplementary Material

Supporting Information
