# Supplementary material for: Global Linkage Map Connects Meiotic Centromere Function to Chromosome Size in Budding Yeast
Source: G3 (Bethesda). 2013 Oct 1;3(10):1741–51. doi: 10.1534/g3.113.007377 (PMC3789798; doi:10.1534/g3.113.007377)
Supplement: Supporting Information [file supp_3_10_1741__index.html]

Global Linkage Map Connects Meiotic Centromere Function to Chromosome Size in Budding Yeast — Supporting Information 

# Global Linkage Map Connects Meiotic Centromere Function to Chromosome Size in Budding Yeast

## Supporting Information for Baryshnikova *et al.*, 2013

**Files in this Data Supplement:**

- Supporting Information - Figures S1-S6, Table S1, File S1, and Supplementary References (PDF, 2 MB)
- Figure S1 - A genetic linkage map for each chromosome was constructed as described in Methods and visualized as a heatmap. (PDF, 1 MB)
- Figure S2 - Examples of genetic linkage profiles of query and array mutants involving the same genomic locus (PDF, 225 KB)
- Figure S3 - Chromosome III carries the yeast mating type locus (*MAT*), which determines the mating type of a yeast haploid cell and distinguishes array and query mutants, which are *MAT*a and *MAT*α, respectively. (PDF, 278 KB)
- Figure S4 - The overlap between any two datasets is visualized as a heatmap/table, where colors and numbers reflect the fraction of hotspots identified by study 1 (row labels) that are located within 10 kb from a hotspot identified by study 2 (column labels). (PDF, 207 KB)
- Figure S5 - Pearson correlation coefficients and the corresponding significance p-values were computed between chromosome size and centromere-proximal recombination reported by several published studies, as described in Methods. (PDF, 159 KB)
- Figure S6 - Relationship between chromosome size and centromere-associated cohesin clustering, measured from data reported previously (Kugou et al. 2009). (PDF, 150 KB)
- Table S1 - Data sources for large-scale recombination and cohesin binding studies (PDF, 266 KB)
- Supplementary References - PDF, 155 KB
- File S1 - SGA-based genetic distances (.zip, 2 MB)
