# Supplementary material for: Global Linkage Map Connects Meiotic Centromere Function to Chromosome Size in Budding Yeast
Source: G3 (Bethesda). 2013 Oct 1;3(10):1741–51. doi: 10.1534/g3.113.007377 (PMC3789798; doi:10.1534/g3.113.007377)
Supplement: Supporting Information [file supp_g3.113.007377_007377SI.pdf]

G3 · Genes | Genomes | Genetics

Global Linkage Map Connects Meiotic Centromere Function to Chromosome Size in Budding Yeast

Anastasia Baryshnikova[§,†,1], Benjamin VanderSluis[‡], Michael Costanzo[§], Chad L. Myers[‡], Rita S. Cha[§§*], Brenda Andrews[§,†,*], Charles Boone[§,†,*]

Affiliations:

[§] Banting and Best Department of Medical Research, The Donnelly Center for Cellular and Biomolecular Research, University of Toronto, Toronto, Ontario M5S 3E1, Canada

[†] Department of Molecular Genetics, University of Toronto, Toronto, Ontario M5S 3E1, Canada

[‡] Department of Computer Science and Engineering, University of Minnesota, Minneapolis, MN 55455, USA

[§§] Department of Life Sciences, Genome Damage and Stability Centre, University of Sussex, Falmer, BN1 9RQ, UK

[1] Current address: Lewis-Sigler Institute for Integrative Genomics, Princeton University, Princeton, NJ 08544, USA

* Correspondence to be addressed to: rc320@sussex.ac.uk, brenda.andrews@utoronto.ca, charlie.boone@utoronto.ca

DOI: http://www.g3journal.org/lookup/suppl/doi:10.1534/g3.113.007377/-/DC1

Figure S1 (A) A genetic linkage map for each chromosome was constructed as described in Methods and visualized as a heatmap. The horizontal and the vertical axes of the heatmap represent chromosomal coordinates; a third dimension indicating the SGA-based genetic distance (SGA-GD) between the corresponding chromosomal regions is represented by the intensity of color of each point in the image. Grey corresponds to missing data due to the experimental constrains of the SGA method (Methods). Each chromosome is drawn to scale. Purple dots represent centromeres. Green dots correspond to the top 10 highest double-strand break hotspots as reported by Pan et al., 2011.

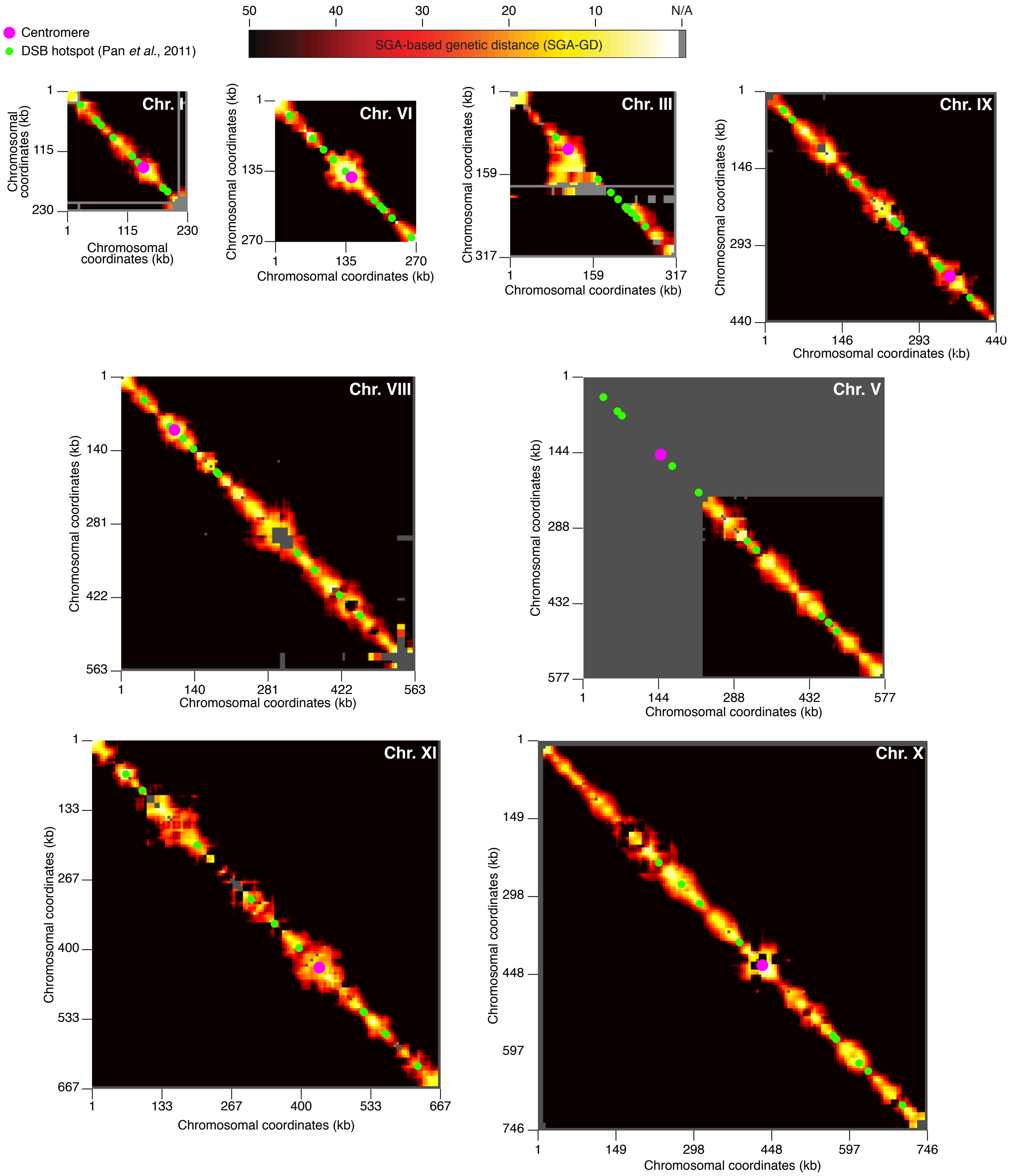

B

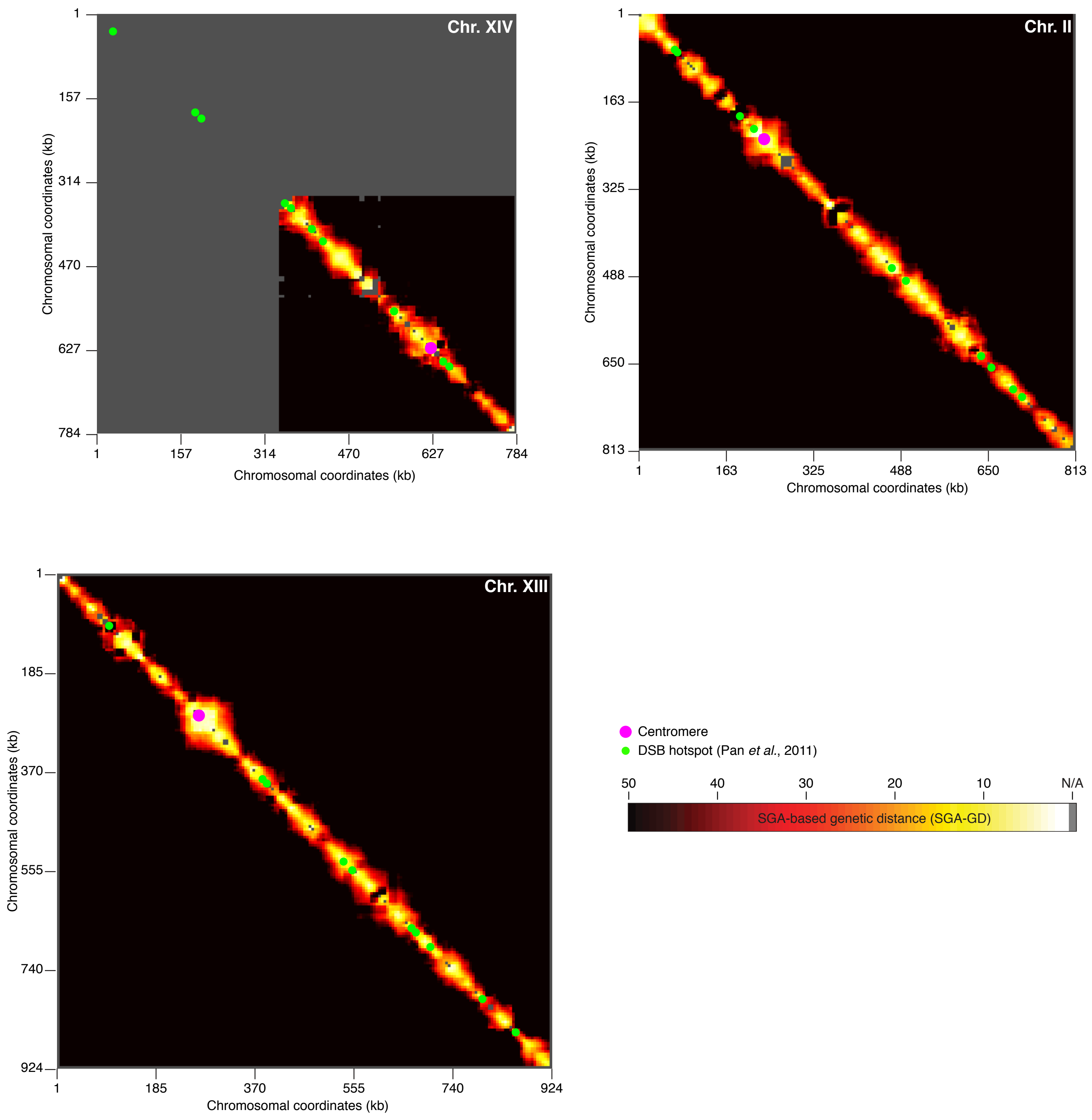
Chr. XIV
Chromosomal coordinates (kb)
1
157
314
470
627
784
Chr. II
1
163
325
488
650
813
Chr. XIII
1
185
370
555
740
924
Centromere
DSB hotspot (Pan et al., 2011)
50
40
30
20
10
N/A
SGA-based genetic distance (SGA-GD)

C

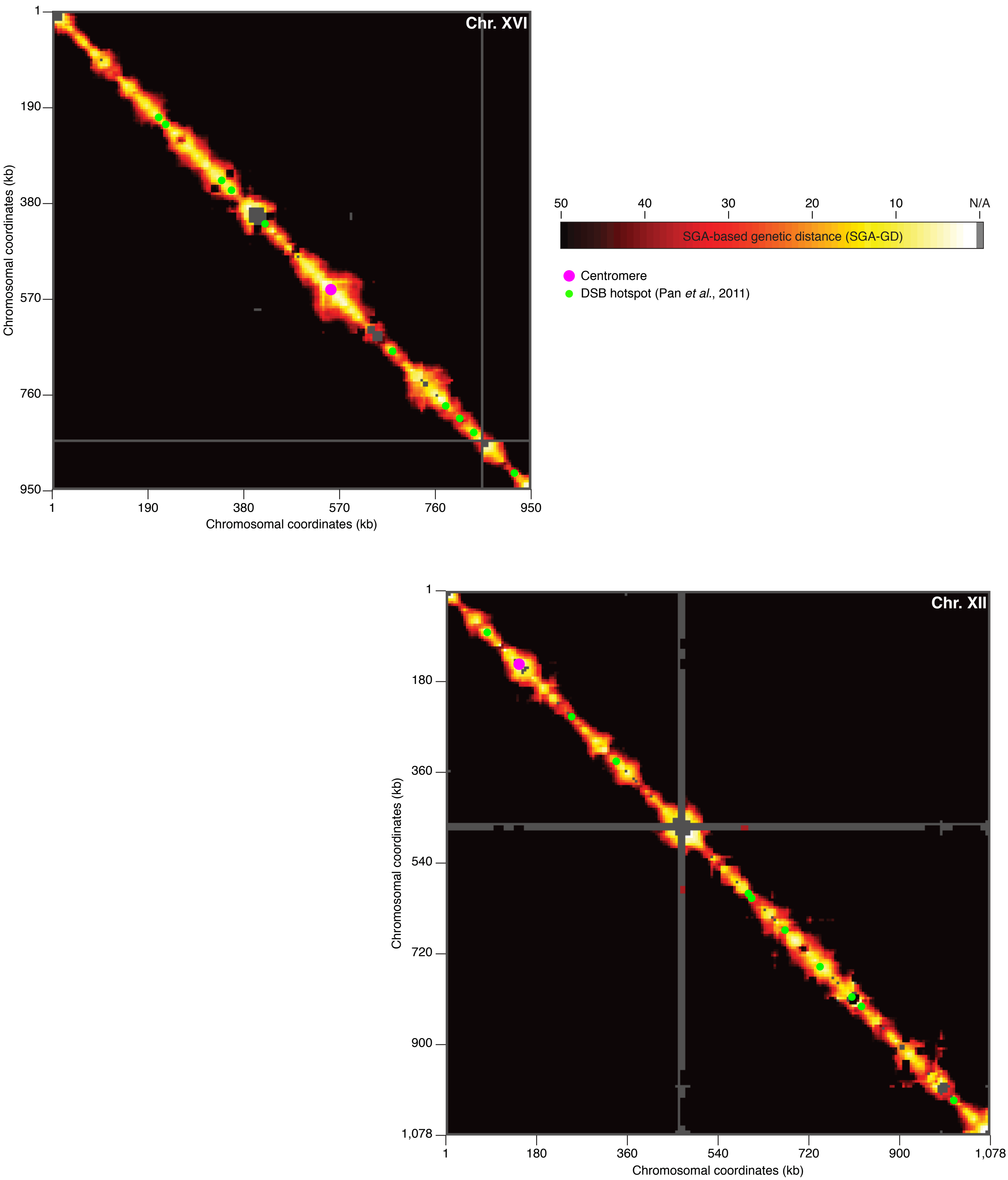

Chr. XVI
1
190
380
570
760
950
Chromosomal coordinates (kb)
50
40
30
20
10
N/A
SGA-based genetic distance (SGA-GD)
Centromere
DSB hotspot (Pan et al., 2011)
Chr. XII
1
180
360
540
720
900
1,078
Chromosomal coordinates (kb)

D

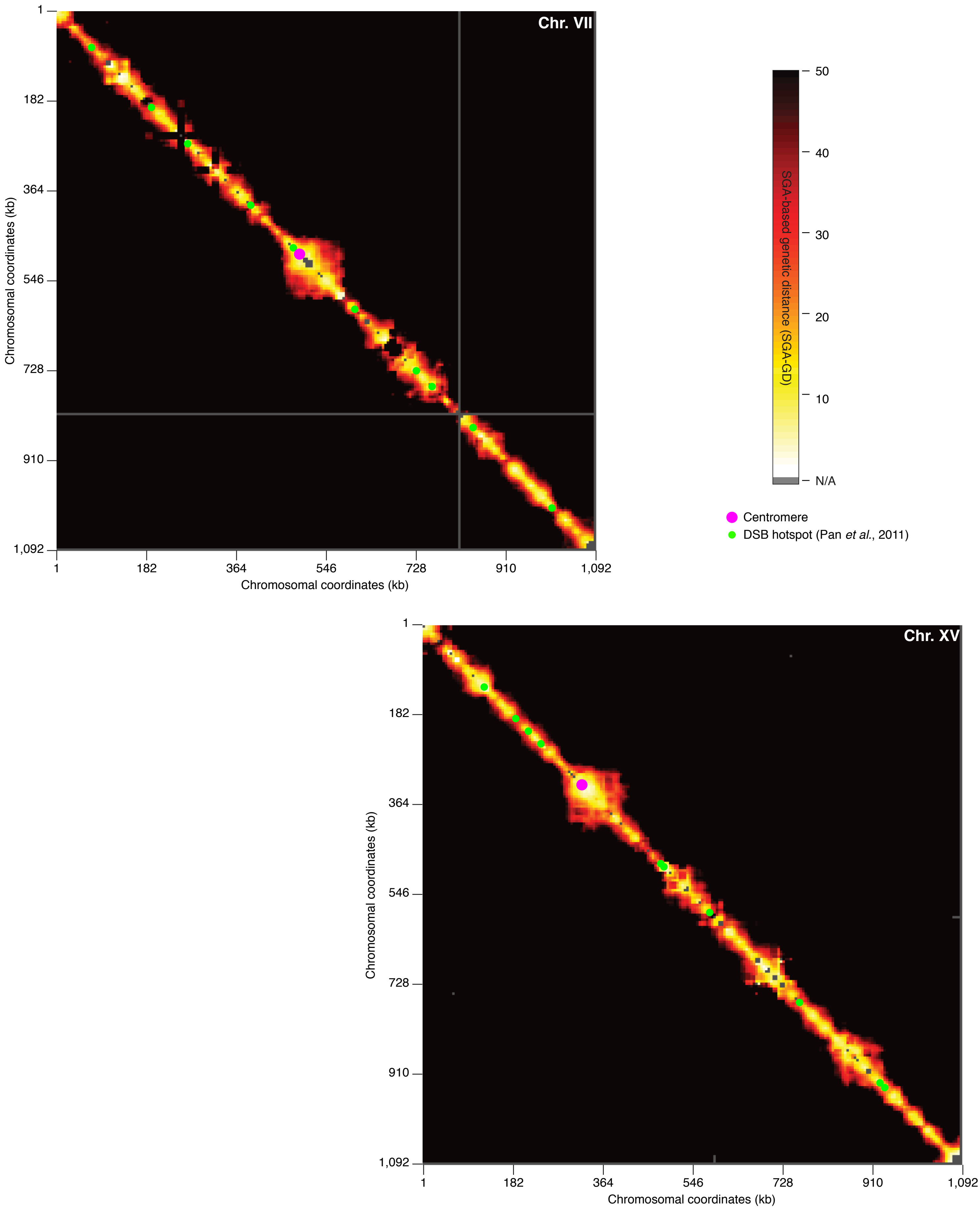
Chr. VII
Chr. XV
Chromosomal coordinates (kb)
Chromosomal coordinates (kb)
1
182
364
546
728
910
1,092
SGA-based genetic distance (SGA-GD)
50
40
30
20
10
N/A
Centromere
DSB hotspot (Pan *et al.*, 2011)

E

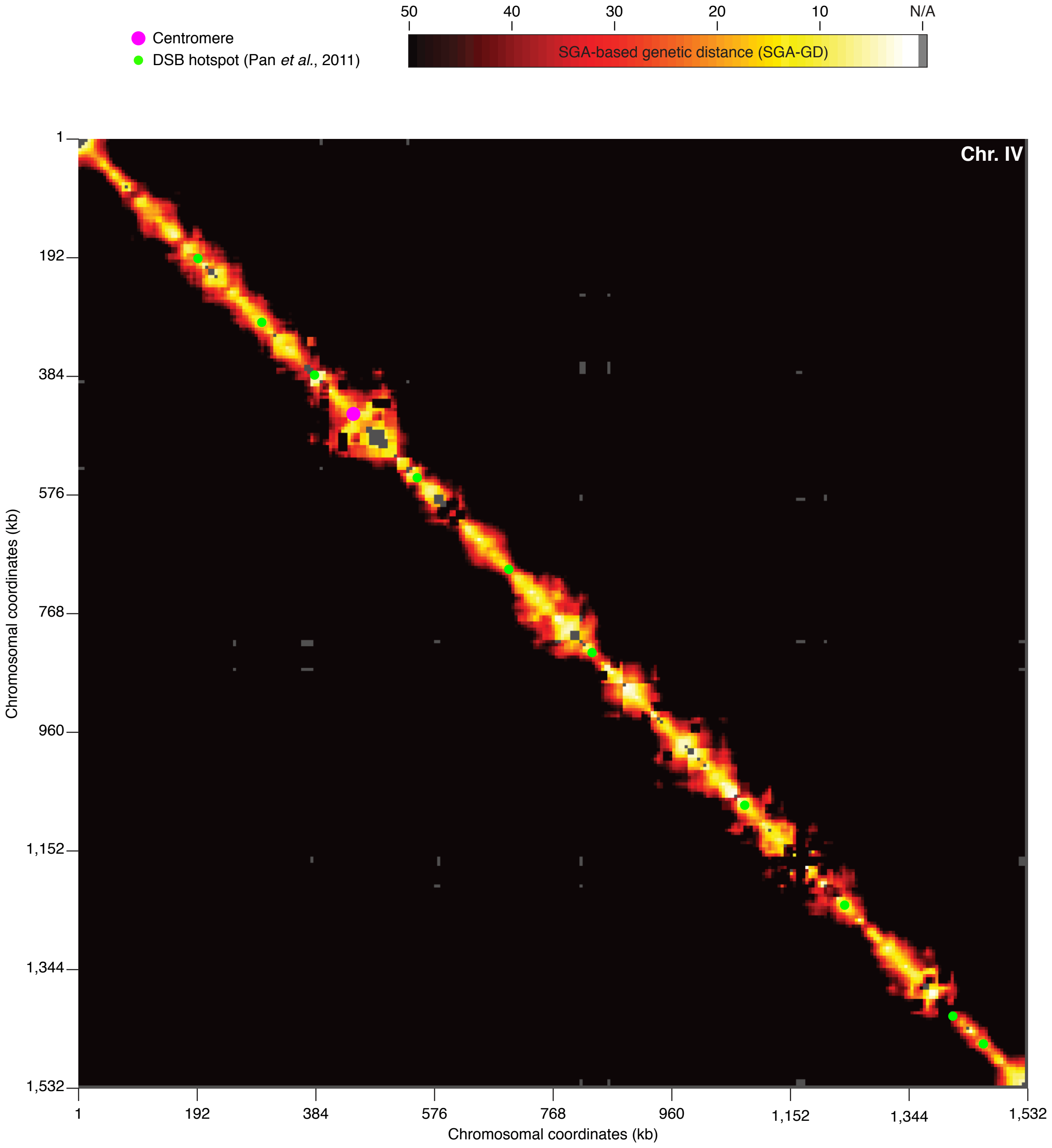

Centromere
DSB hotspot (Pan *et al.*, 2011)
50
40
30
20
10
N/A
SGA-based genetic distance (SGA-GD)
Chr. IV
1
192
384
576
768
960
1,152
1,344
1,532
Chromosomal coordinates (kb)
Chromosomal coordinates (kb)

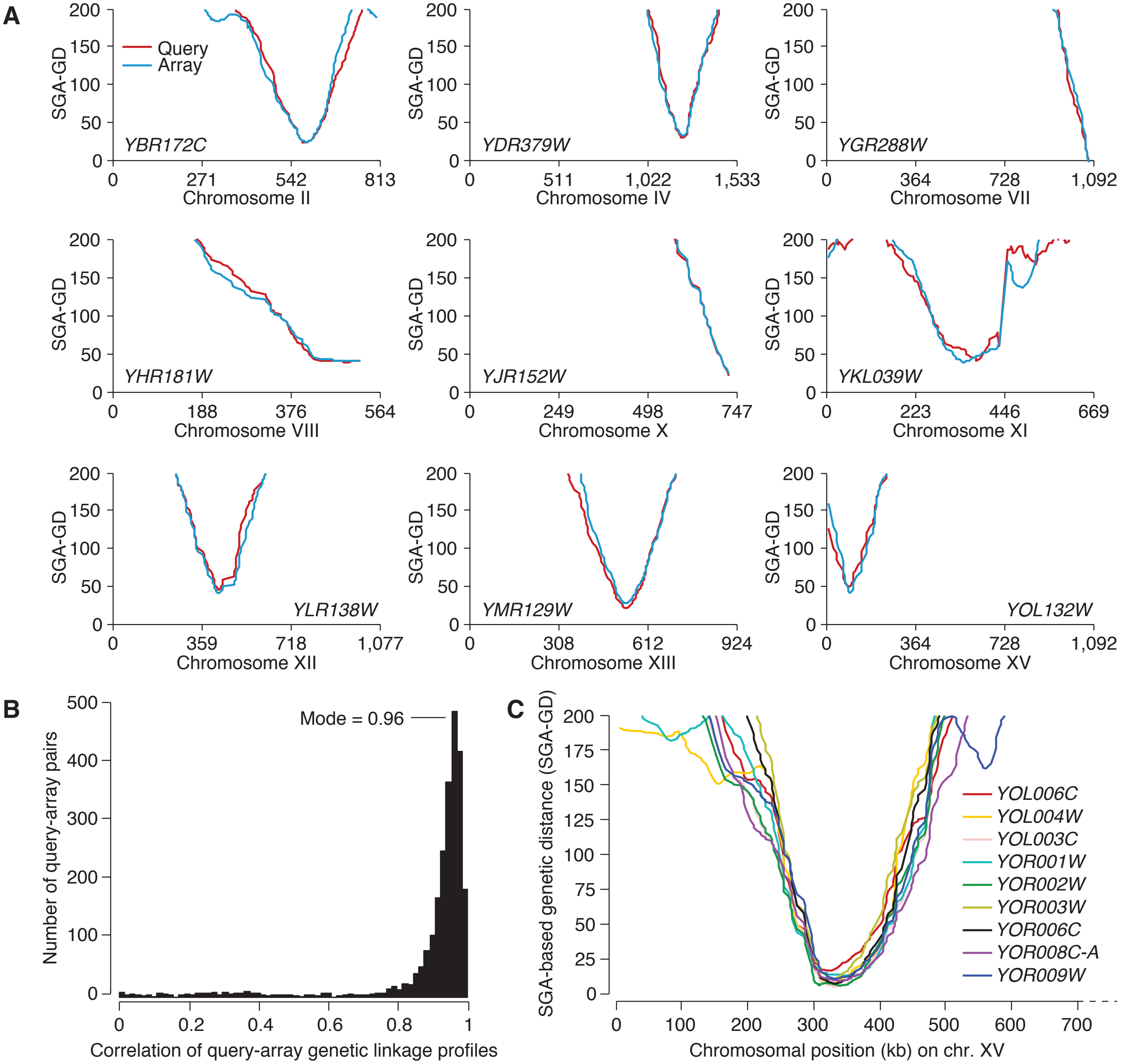

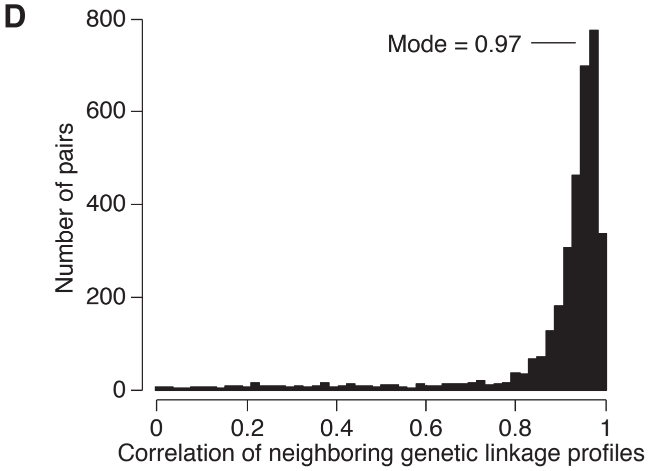

Figure S2 (A) Examples of genetic linkage profiles of query and array mutants involving the same genomic locus. A genetic linkage profile is defined as the set of genetic distances, measured in SGA-GD (Methods), between a given locus and all other loci present on the same chromosome. The reproducibility of genetic linkage profiles is assessed by systematically comparing two independent experiments: in one experiment, the locus of interest has been deleted using the *natMX4* selectable marker ("query" mutant); in the other experiment, the locus of interest has been deleted using the *kanMX* selectable marker ("array" mutant). **(B)** Distribution of Pearson correlation coefficients among the genetic linkage profiles of ~2,800 query-array mutant pairs. Each pair corresponds to two independent experiments for a given genomic locus. **(C)** Genetic linkage profiles for 9 consecutive genes located on chromosome XV. Consecutive loci are expected to have similar genetic linkage profiles because they are likely to experience similar local recombination rates. **(D)** Distribution of Pearson correlation coefficients for ~3,700 pairs of genetic linkage profiles involving consecutive genomic loci.

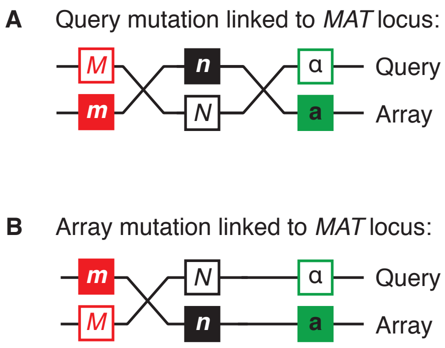

Figure S3 Chromosome III carries the yeast mating type locus (*MAT*), which determines the mating type of a yeast haploid cell and distinguishes array and query mutants, which are *MAT***a** and *MAT*α, respectively. Since SGA selects specifically for *MAT***a** meiotic progeny, a query mutant *n* (**A**, filled black square), located between gene *M* (**A**, empty red square) and the *MAT* locus (**A**, empty green square), requires a double recombination event to acquire both the array mutation *m* (**A**, filled red square) and the *MAT***a** information (**A**, filled green square). The array mutant *n*, however, (**B**, filled black square), being already linked to *MAT***a**, only requires one recombination event to acquire query mutation *m* (**B**, filled red square). As a result, query and array mutants linked to the *MAT* locus show different degrees of linkage to nearby genes, and averaging their genetic linkage maps would conceal the true recombination activity occurring within the region.

Study 2 (columns); **Study 1** (rows)

Study 1 \ Study 2	Gerton et al., 2000	Borde *et al.*, 2004	Buhler *et al.*, 2007	Blitzblau *et al.*, 2007	Pan *et al.*, 2011	Mancera *et al.*, 2008	This study	Average precision
Gerton et al., 2000		0.77	0.68	0.60	0.85	0.55	0.72	0.68
Borde *et al.*, 2004	0.53		0.78	0.53	0.79	0.43	0.67	0.63
Buhler *et al.*, 2007	0.51	0.84		0.57	0.84	0.39	0.66	0.63
Blitzblau *et al.*, 2007	0.45	0.57	0.56		0.85	0.43	0.62	0.55
Pan *et al.*, 2011	0.46	0.66	0.62	0.61		0.40	0.65	0.55
Mancera *et al.*, 2008	0.58	0.62	0.59	0.68	0.73		0.77	0.62
This study	0.35	0.52	0.40	0.36	0.57	0.34		0.41
Average sensitivity	0.50	0.70	0.64	0.57	0.79	0.44	0.68	

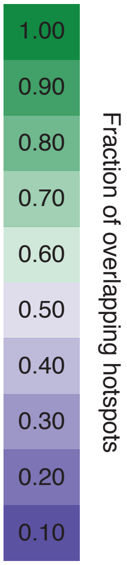

Figure S4 The overlap between any two datasets is visualized as a heatmap/table, where colors and numbers reflect the fraction of hotspots identified by study 1 (row labels) that are located within 10 kb from a hotspot identified by study 2 (column labels). For example, of all hotspots identified by Gerton *et al.* (row label), 77% were also identified by Borde *et al.* (column label), and, vice versa, of all hotspots identified by Borde *et al.* (row label), 53% were also identified by Gerton *et al.* (column label).

Significant								
Not significant								

	Centromere linkage				Overall linkage (control)			
	All chromosomes		All chrs., excl. 4 smallest		All chromosomes		All chrs., excl. 4 smallest	
	R	*p-val*	*R*	*p-val*	*R*	*p-val*	*R*	*p-val*
This study	0.77	0.01	0.66	0.03	0.74	0.001	0.44	0.15
SGD	0.70	0.002	0.42	0.17	0.64	0.008	0.05	0.87
Gerton *et al.*, 2000	0.78	0.0003	0.74	0.006	0.46	0.08	–0.07	0.82
Borde *et al.*, 2004	0.79	0.001	0.92	0.0002	0.25	0.34	0.04	0.89
Buhler *et al.*, 2007	0.63	0.04	0.72	0.03	0.33	0.22	0.15	0.64
Blitzblau *et al.*, 2007	0.59	0.02	0.67	0.02	0.62	0.01	0.11	0.74

Figure S5 Pearson correlation coefficients and the corresponding significance p-values were computed between chromosome size and centromere-proximal recombination reported by several published studies, as described in Methods.

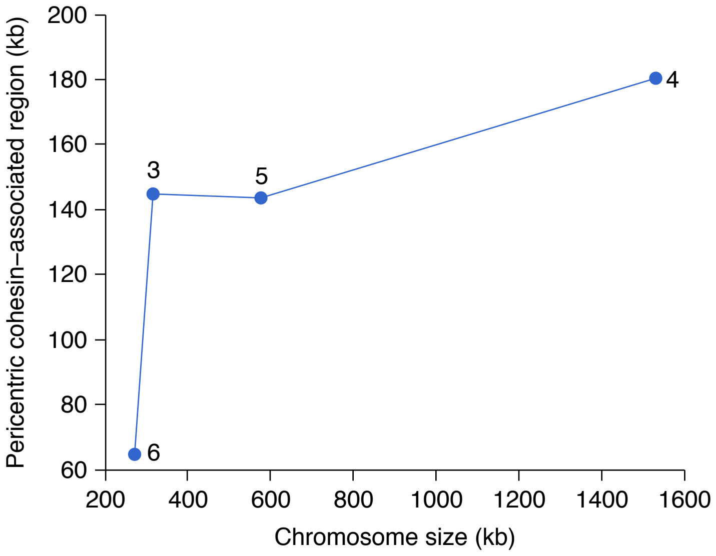

Figure S6 Relationship between chromosome size and centromere-associated cohesin clustering, measured from data reported previously (Kugou et al. 2009). Data relative to 1.5 hours after induction of meiosis are shown. Each data point corresponds to a chromosome, labeled by the number. Pearson correlation coefficient is $R = 0.73$ but not significant (p-value < 0.27).

Table S1 Data sources for large-scale recombination and cohesin binding studies

**Publication**	**Data source**
(Gerton *et al.* 2000)	Table 1
(Borde *et al.* 2004)	Table S2
(Buhler *et al.* 2007)	Table S5 (5X background)
(Blitzblau *et al.* 2007)	Table S2
(Mancera *et al.* 2008)	Supplementary information 2: hot_spots.txt
(Pan *et al.* 2011)	Table S2 (signal intensity > 1000)
(Glynn *et al.* 2004)	Mitotic cohesin subunit (Scc1/Mcd1): Supplementary dataset 8<br>Meiotic cohesin subunit (Rec8): Supplementary dataset 9
(Kiburz *et al.* 2005)	Supp Raw Data.doc
(Kugou *et al.* 2009)	http://www.ncbi.nlm.nih.gov/geo/query/acc.cgi?acc=GSE8422

File S1

SGA-based genetic distances

Available for download at http://www.g3journal.org/lookup/suppl/doi:10.1534/g3.113.007377/-/DC1

A set of 16 files, one for each chromosome, contain the SGA-based genetic distances (SGA-GD; Methods) for the indicated loci. Rows and columns correspond to query and array mutants, respectively. Multiple experiments, involving the same query locus, appear as duplicated rows in each file.

Supplementary references

Anscombe, F. J., 1973 Graphs in Statistical Analysis. The American Statistician **27:** 17-21.

Bishop, D. K., and D. Zickler, 2004 Early decision; meiotic crossover interference prior to stable strand exchange and synapsis. Cell **117:** 9-15.

Blitzblau, H. G., G. W. Bell, J. Rodriguez, S. P. Bell and A. Hochwagen, 2007 Mapping of meiotic single-stranded DNA reveals double-stranded-break hotspots near centromeres and telomeres. Current biology : CB **17:** 2003-2012.

Borde, V., W. Lin, E. Novikov, J. H. Petrini, M. Lichten *et al.*, 2004 Association of Mre11p with double-strand break sites during yeast meiosis. Molecular cell **13:** 389-401.

Buhler, C., V. Borde and M. Lichten, 2007 Mapping meiotic single-strand DNA reveals a new landscape of DNA double-strand breaks in Saccharomyces cerevisiae. PLoS biology **5:** e324.

Cherry, J. M., E. L. Hong, C. Amundsen, R. Balakrishnan, G. Binkley *et al.*, 2012 Saccharomyces Genome Database: the genomics resource of budding yeast. Nucleic acids research **40:** D700-705.

Gerton, J. L., J. Derisi, R. Shroff, M. Lichten, P. O. Brown *et al.*, 2000 Global mapping of meiotic recombination hotspots and coldspots in the yeast Saccharomyces cerevisiae. Proceedings of the National Academy of Sciences of the United States of America **97:** 11383-11390.

Glynn, E. F., P. C. Megee, H. G. Yu, C. Mistrot, E. Unal *et al.*, 2004 Genome-wide mapping of the cohesin complex in the yeast Saccharomyces cerevisiae. PLoS biology **2:** E259.

Jones, G. H., 1987 Chiasmata, pp. 213-244 in *Meiosis*, edited by P. B. MOENS. Academic Press, Orlando, FL.

Kaback, D. B., D. Barber, J. Mahon, J. Lamb and J. You, 1999 Chromosome size-dependent control of meiotic reciprocal recombination in Saccharomyces cerevisiae: the role of crossover interference. Genetics **152:** 1475-1486.

Kaback, D. B., V. Guacci, D. Barber and J. W. Mahon, 1992 Chromosome size-dependent control of meiotic recombination. Science **256:** 228-232.

Kiburz, B. M., D. B. Reynolds, P. C. Megee, A. L. Marston, B. H. Lee *et al.*, 2005 The core centromere and Sgo1 establish a 50-kb cohesin-protected domain around centromeres during meiosis I. Genes & development **19:** 3017-3030.

Kugou, K., T. Fukuda, S. Yamada, M. Ito, H. Sasanuma *et al.*, 2009 Rec8 guides canonical Spo11 distribution along yeast meiotic chromosomes. Mol Biol Cell **20:** 3064-3076.

Mancera, E., R. Bourgon, A. Brozzi, W. Huber and L. M. Steinmetz, 2008 High-resolution mapping of meiotic crossovers and non-crossovers in yeast. Nature **454:** 479-485.

Pan, J., M. Sasaki, R. Kniewel, H. Murakami, H. G. Blitzblau *et al.*, 2011 A hierarchical combination of factors shapes the genome-wide topography of yeast meiotic recombination initiation. Cell **144:** 719-731.

Pinsky, B. A., and S. Biggins, 2005 The spindle checkpoint: tension versus attachment. Trends in cell biology **15:** 486-493.

Stahl, F. W., H. M. Foss, L. S. Young, R. H. Borts, M. F. Abdullah *et al.*, 2004 Does crossover interference count in Saccharomyces cerevisiae? Genetics **168:** 35-48.
