# Supplementary material for: Global Linkage Map Connects Meiotic Centromere Function to Chromosome Size in Budding Yeast
Source: G3 (Bethesda). 2013 Oct 1;3(10):1741–51. doi: 10.1534/g3.113.007377 (PMC3789798; doi:10.1534/g3.113.007377)
Supplement: Supporting Information [file supp_g3.113.007377_FigureS1.pdf]

Figure S1 (A) A genetic linkage map for each chromosome was constructed as described in Methods and visualized as a heatmap. The horizontal and the vertical axes of the heatmap represent chromosomal coordinates; a third dimension indicating the SGA-based genetic distance (SGA-GD) between the corresponding chromosomal regions is represented by the intensity of color of each point in the image. Grey corresponds to missing data due to the experimental constrains of the SGA method (Methods). Each chromosome is drawn to scale. Purple dots represent centromeres. Green dots correspond to the top 10 highest double-strand break hotspots as reported by Pan et al., 2011.

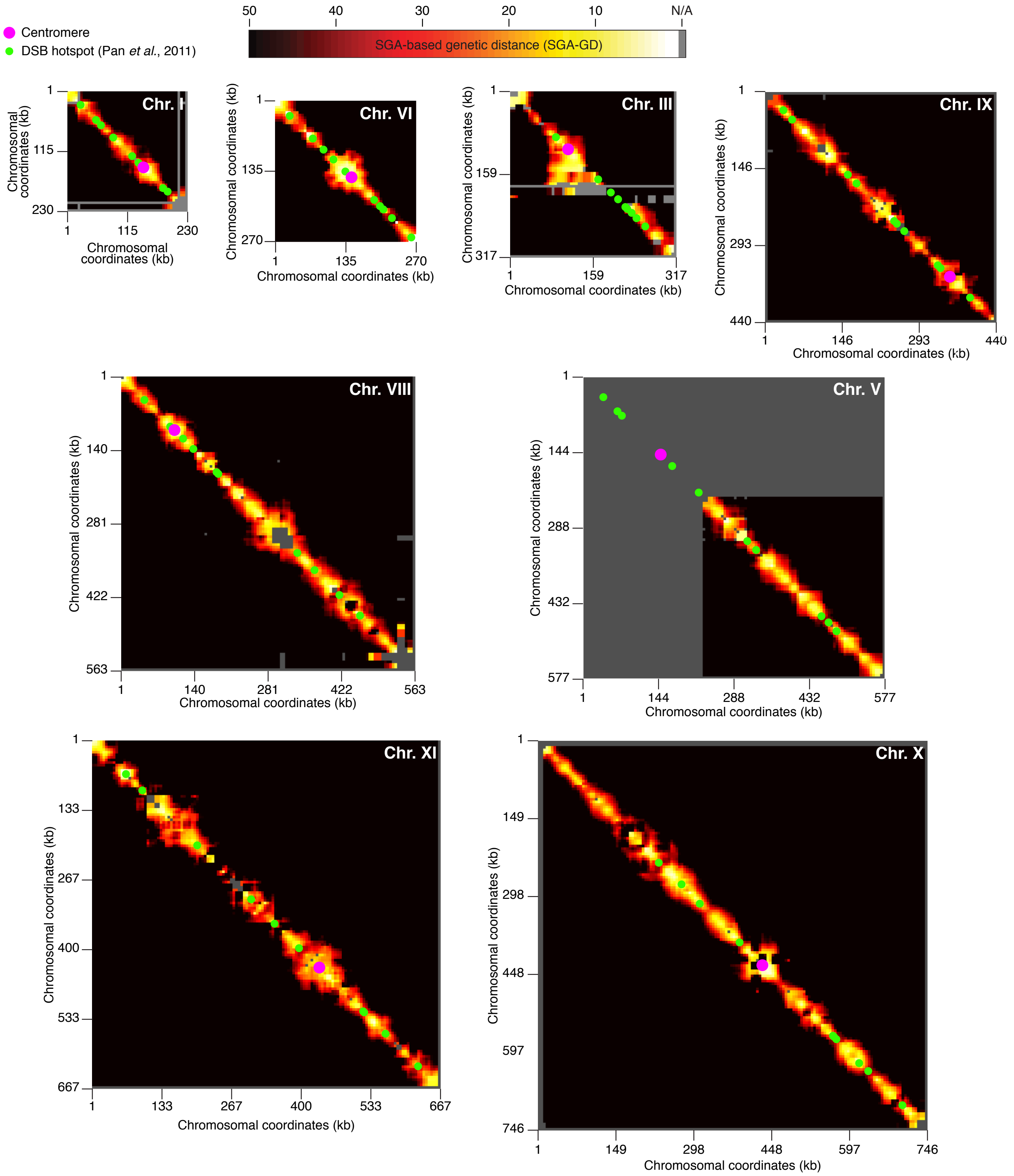

B

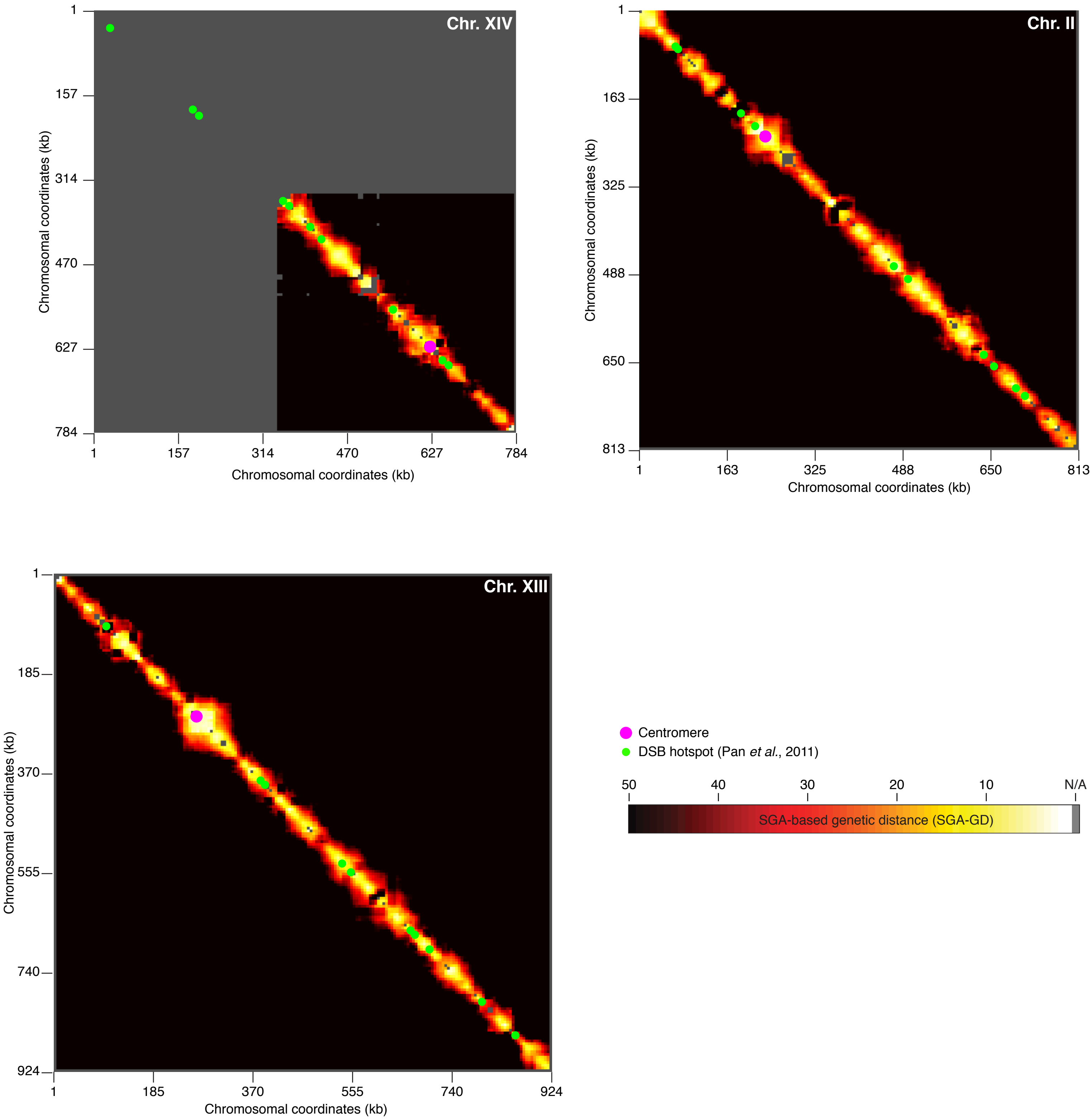
Chr. XIV
Chromosomal coordinates (kb)
1
157
314
470
627
784
Chromosomal coordinates (kb)
Chr. II
Chromosomal coordinates (kb)
1
163
325
488
650
813
Chromosomal coordinates (kb)
Chr. XIII
Chromosomal coordinates (kb)
1
185
370
555
740
924
Chromosomal coordinates (kb)
Centromere
DSB hotspot (Pan *et al.*, 2011)
50
40
30
20
10
N/A
SGA-based genetic distance (SGA-GD)

C

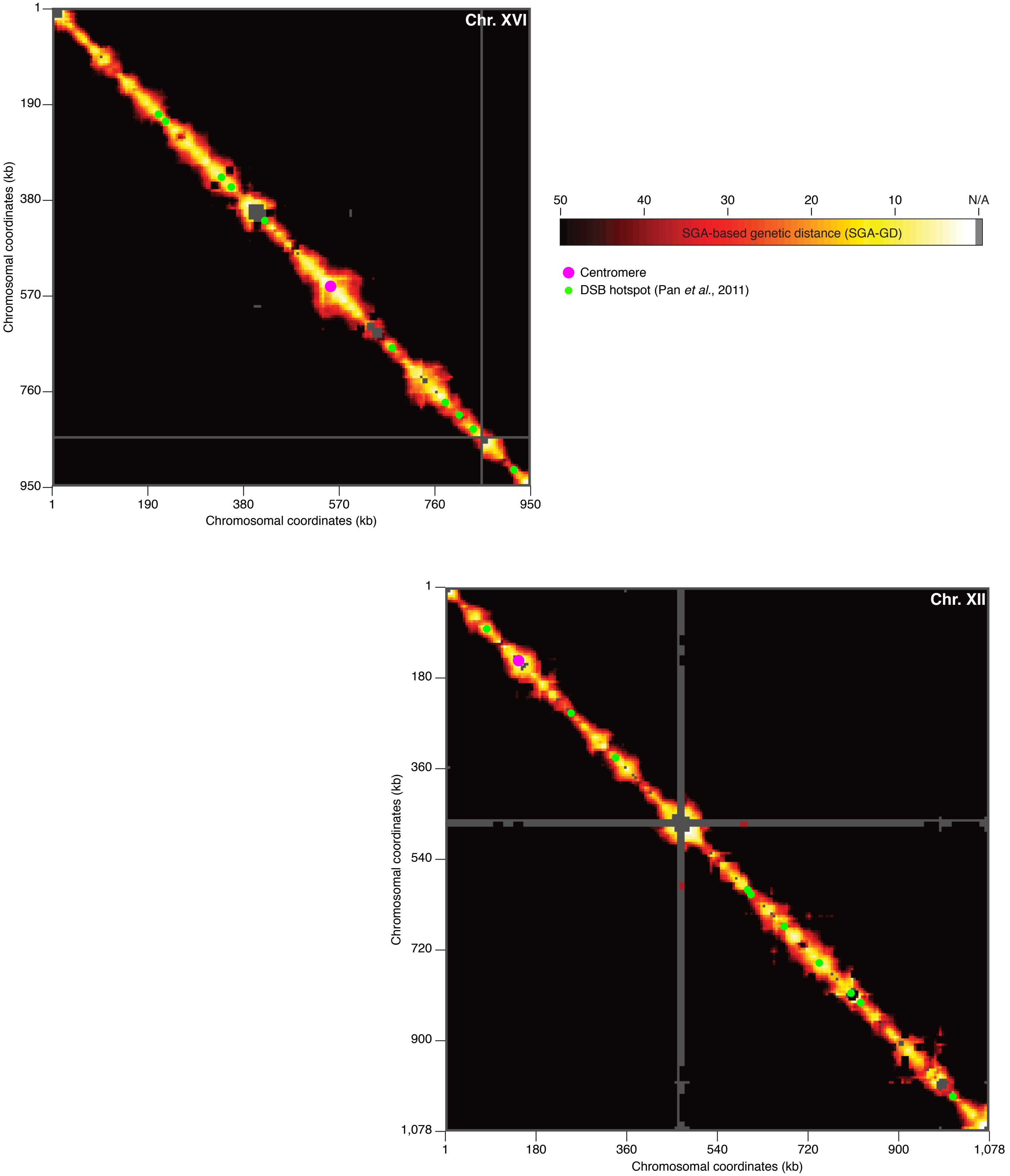

Chr. XVI
Chromosomal coordinates (kb)
1
190
380
570
760
950
50
40
30
20
10
N/A
SGA-based genetic distance (SGA-GD)
Centromere
DSB hotspot (Pan et al., 2011)
Chr. XII
180
360
540
720
900
1,078

D

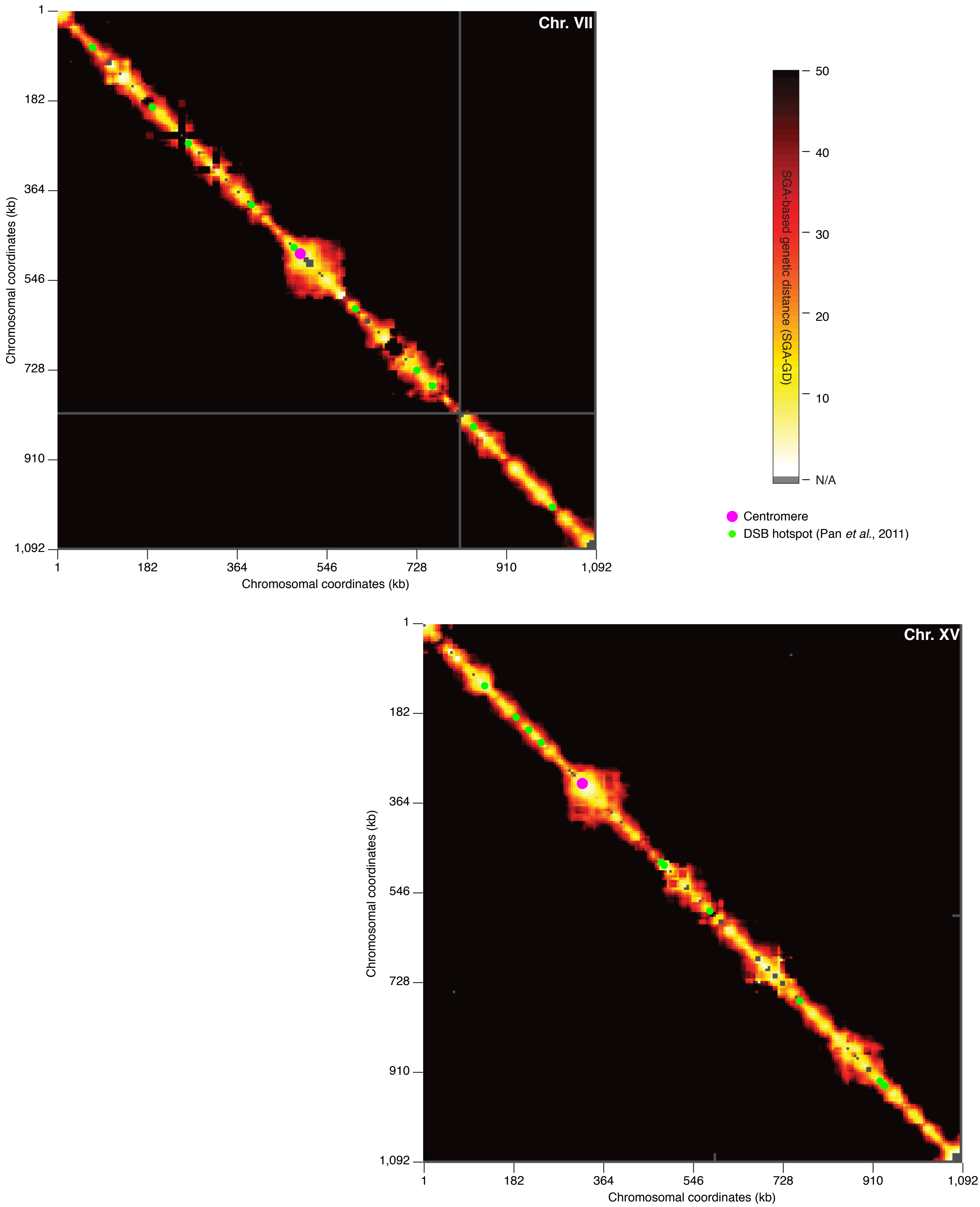
Chr. VII
1
182
364
546
728
910
1,092
Chromosomal coordinates (kb)
50
40
30
20
10
N/A
SGA-based genetic distance (SGA-GD)
Centromere
DSB hotspot (Pan *et al.*, 2011)
Chr. XV

E

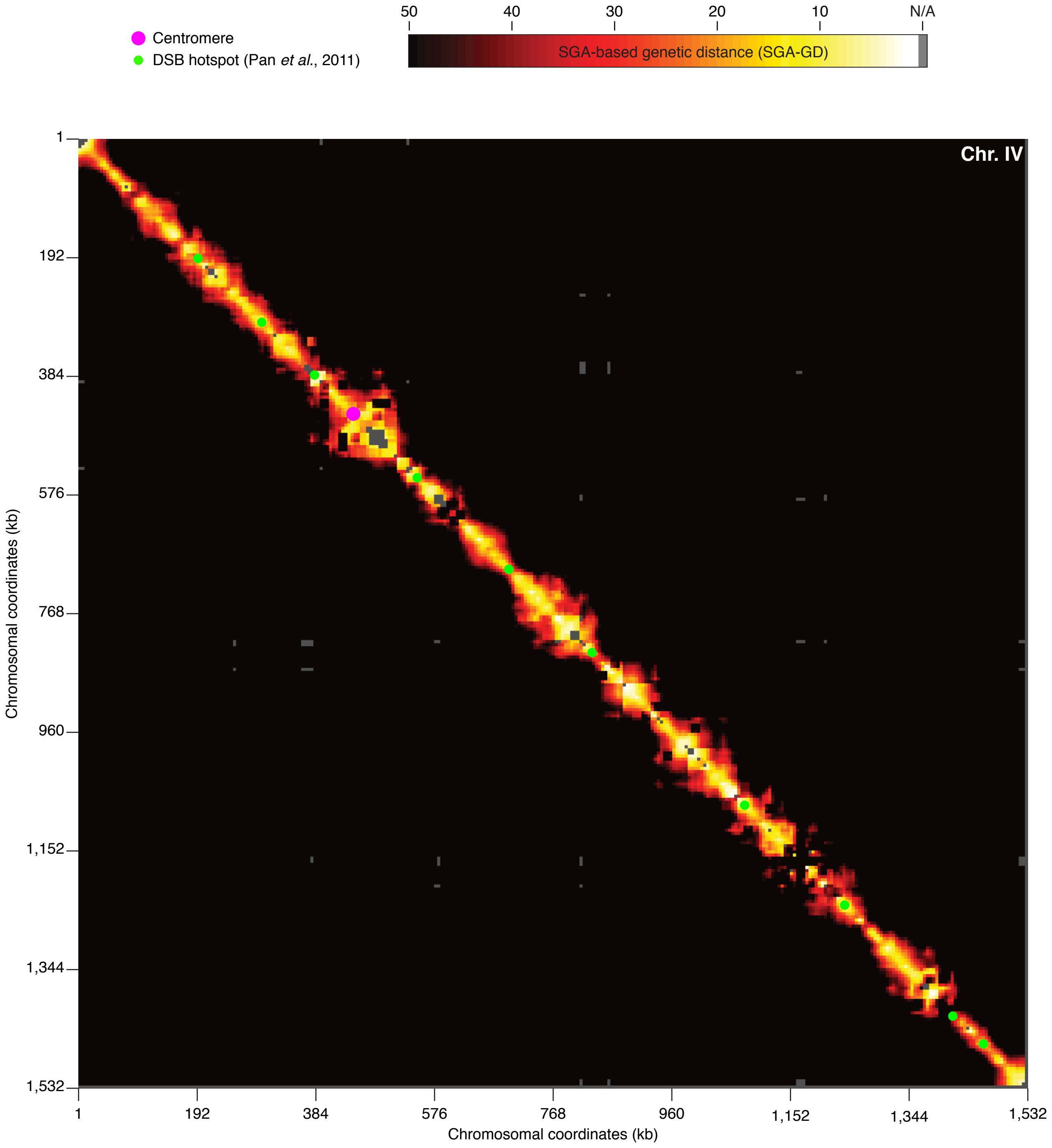
Centromere
DSB hotspot (Pan *et al.*, 2011)
50
40
30
20
10
N/A
SGA-based genetic distance (SGA-GD)
Chr. IV
Chromosomal coordinates (kb)
1
192
384
576
768
960
1,152
1,344
1,532
Chromosomal coordinates (kb)
