# Supplementary material for: Global Linkage Map Connects Meiotic Centromere Function to Chromosome Size in Budding Yeast
Source: G3 (Bethesda). 2013 Oct 1;3(10):1741–51. doi: 10.1534/g3.113.007377 (PMC3789798; doi:10.1534/g3.113.007377)
Supplement: Supporting Information [file supp_g3.113.007377_FigureS2.pdf]

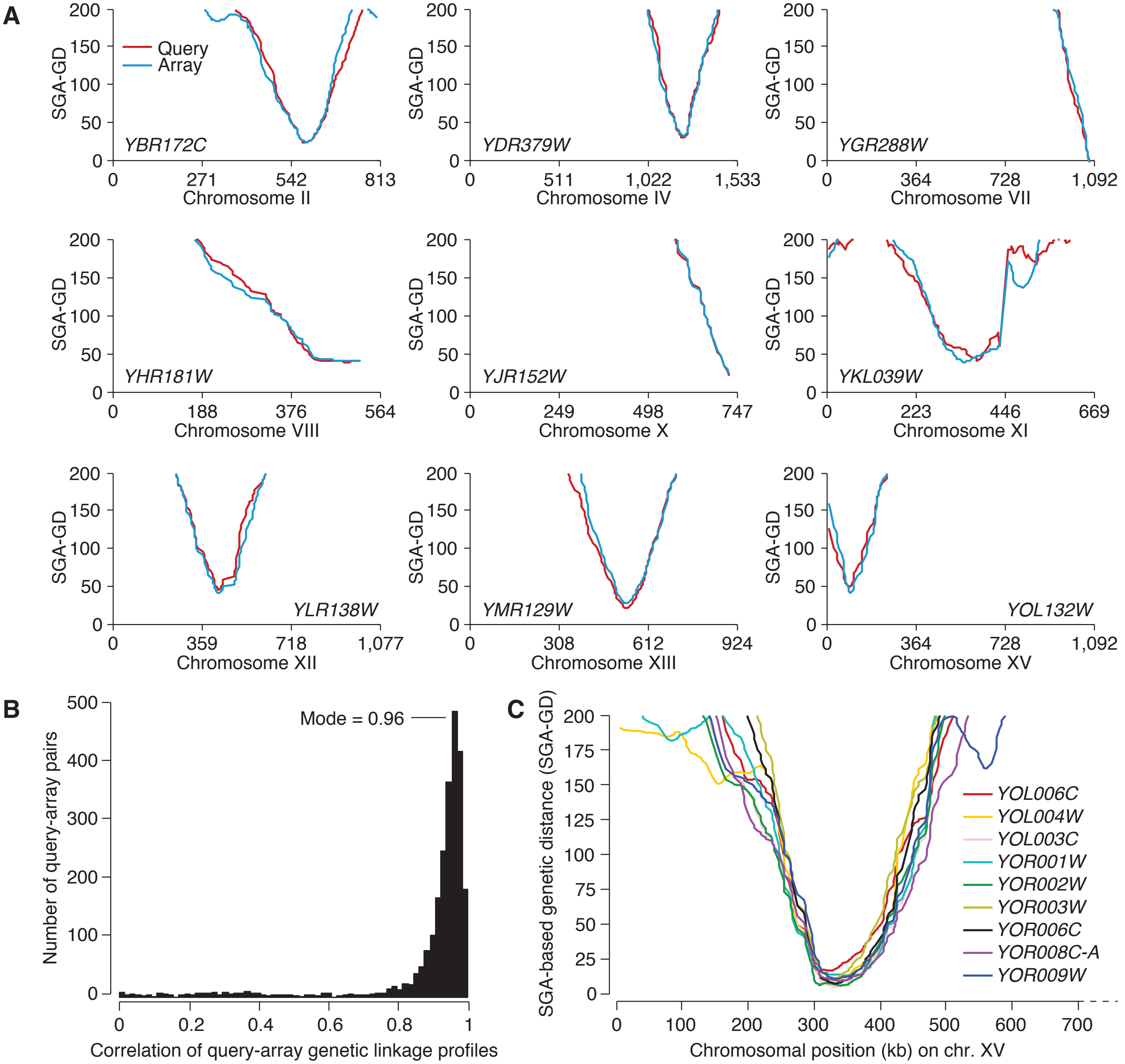

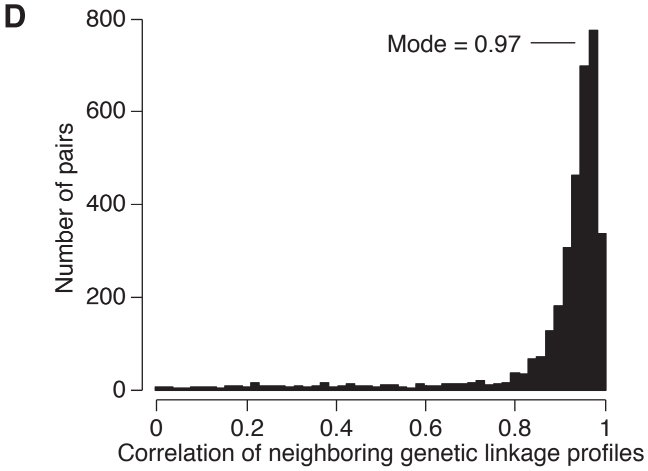

Figure S2 (A) Examples of genetic linkage profiles of query and array mutants involving the same genomic locus. A genetic linkage profile is defined as the set of genetic distances, measured in SGA-GD (Methods), between a given locus and all other loci present on the same chromosome. The reproducibility of genetic linkage profiles is assessed by systematically comparing two independent experiments: in one experiment, the locus of interest has been deleted using the *natMX4* selectable marker ("query" mutant); in the other experiment, the locus of interest has been deleted using the *kanMX* selectable marker ("array" mutant). **(B)** Distribution of Pearson correlation coefficients among the genetic linkage profiles of ~2,800 query-array mutant pairs. Each pair corresponds to two independent experiments for a given genomic locus. **(C)** Genetic linkage profiles for 9 consecutive genes located on chromosome XV. Consecutive loci are expected to have similar genetic linkage profiles because they are likely to experience similar local recombination rates. **(D)** Distribution of Pearson correlation coefficients for ~3,700 pairs of genetic linkage profiles involving consecutive genomic loci.
