# Supplementary material for: Global Linkage Map Connects Meiotic Centromere Function to Chromosome Size in Budding Yeast
Source: G3 (Bethesda). 2013 Oct 1;3(10):1741–51. doi: 10.1534/g3.113.007377 (PMC3789798; doi:10.1534/g3.113.007377)
Supplement: Supporting Information [file supp_g3.113.007377_FigureS3.pdf]

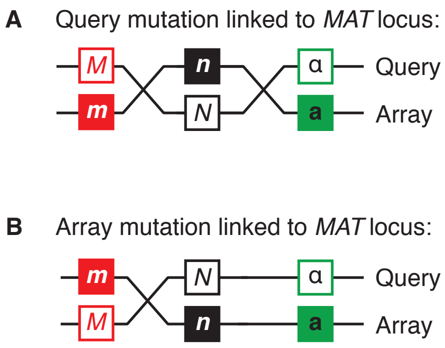

Figure S3 Chromosome III carries the yeast mating type locus (*MAT*), which determines the mating type of a yeast haploid cell and distinguishes array and query mutants, which are *MAT***a** and *MAT*α, respectively. Since SGA selects specifically for *MAT***a** meiotic progeny, a query mutant *n* (**A**, filled black square), located between gene *M* (**A**, empty red square) and the *MAT* locus (**A**, empty green square), requires a double recombination event to acquire both the array mutation *m* (**A**, filled red square) and the *MAT***a** information (**A**, filled green square). The array mutant *n*, however, (**B**, filled black square), being already linked to *MAT***a**, only requires one recombination event to acquire query mutation *m* (**B**, filled red square). As a result, query and array mutants linked to the *MAT* locus show different degrees of linkage to nearby genes, and averaging their genetic linkage maps would conceal the true recombination activity occurring within the region.
