# Supplementary material for: Global Linkage Map Connects Meiotic Centromere Function to Chromosome Size in Budding Yeast
Source: G3 (Bethesda). 2013 Oct 1;3(10):1741–51. doi: 10.1534/g3.113.007377 (PMC3789798; doi:10.1534/g3.113.007377)
Supplement: Supporting Information [file supp_g3.113.007377_FigureS4.pdf]

Study 2 (columns) / **Study 1** (rows)

Study 1 \ Study 2	Gerton et al., 2000	Borde *et al.*, 2004	Buhler *et al.*, 2007	Blitzblau *et al.*, 2007	Pan *et al.*, 2011	Mancera *et al.*, 2008	This study	Average precision
Gerton et al., 2000		0.77	0.68	0.60	0.85	0.55	0.72	0.68
Borde *et al.*, 2004	0.53		0.78	0.53	0.79	0.43	0.67	0.63
Buhler *et al.*, 2007	0.51	0.84		0.57	0.84	0.39	0.66	0.63
Blitzblau *et al.*, 2007	0.45	0.57	0.56		0.85	0.43	0.62	0.55
Pan *et al.*, 2011	0.46	0.66	0.62	0.61		0.40	0.65	0.55
Mancera *et al.*, 2008	0.58	0.62	0.59	0.68	0.73		0.77	0.62
This study	0.35	0.52	0.40	0.36	0.57	0.34		0.41
Average sensitivity	0.50	0.70	0.64	0.57	0.79	0.44	0.68	

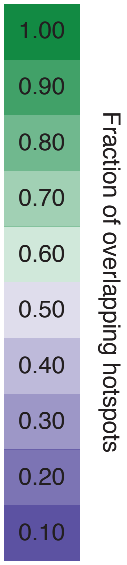

Figure S4 The overlap between any two datasets is visualized as a heatmap/table, where colors and numbers reflect the fraction of hotspots identified by study 1 (row labels) that are located within 10 kb from a hotspot identified by study 2 (column labels). For example, of all hotspots identified by Gerton *et al.* (row label), 77% were also identified by Borde *et al.* (column label), and, vice versa, of all hotspots identified by Borde *et al.* (row label), 53% were also identified by Gerton *et al.* (column label).
