# Supplementary material for: Global Linkage Map Connects Meiotic Centromere Function to Chromosome Size in Budding Yeast
Source: G3 (Bethesda). 2013 Oct 1;3(10):1741–51. doi: 10.1534/g3.113.007377 (PMC3789798; doi:10.1534/g3.113.007377)
Supplement: Supporting Information [file supp_g3.113.007377_FigureS5.pdf]

Significant / Not significant	Centromere linkage				Overall linkage (control)			
	All chromosomes		All chrs., excl. 4 smallest		All chromosomes		All chrs., excl. 4 smallest	
	R	*p-val*	*R*	*p-val*	*R*	*p-val*	*R*	*p-val*
This study	0.77	0.01	0.66	0.03	0.74	0.001	0.44	0.15
SGD	0.70	0.002	0.42	0.17	0.64	0.008	0.05	0.87
Gerton *et al.*, 2000	0.78	0.0003	0.74	0.006	0.46	0.08	–0.07	0.82
Borde *et al.*, 2004	0.79	0.001	0.92	0.0002	0.25	0.34	0.04	0.89
Buhler *et al.*, 2007	0.63	0.04	0.72	0.03	0.33	0.22	0.15	0.64
Blitzblau *et al.*, 2007	0.59	0.02	0.67	0.02	0.62	0.01	0.11	0.74

Figure S5 Pearson correlation coefficients and the corresponding significance p-values were computed between chromosome size and centromere-proximal recombination reported by several published studies, as described in Methods.
