# Supplementary material for: Global Linkage Map Connects Meiotic Centromere Function to Chromosome Size in Budding Yeast
Source: G3 (Bethesda). 2013 Oct 1;3(10):1741–51. doi: 10.1534/g3.113.007377 (PMC3789798; doi:10.1534/g3.113.007377)
Supplement: Supporting Information [file supp_g3.113.007377_FigureS6.pdf]

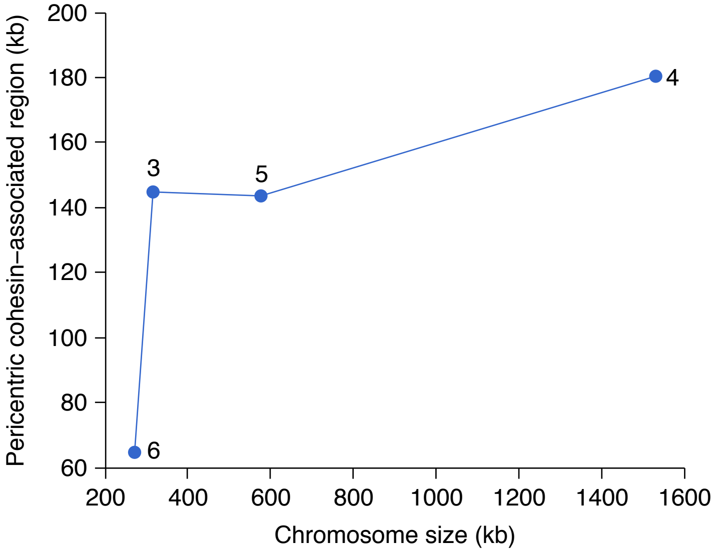

Figure S6 Relationship between chromosome size and centromere-associated cohesin clustering, measured from data reported previously (Kugou et al. 2009). Data relative to 1.5 hours after induction of meiosis are shown. Each data point corresponds to a chromosome, labeled by the number. Pearson correlation coefficient is $R = 0.73$ but not significant (p-value < 0.27).
