# Supplementary material for: Global Linkage Map Connects Meiotic Centromere Function to Chromosome Size in Budding Yeast
Source: G3 (Bethesda). 2013 Oct 1;3(10):1741–51. doi: 10.1534/g3.113.007377 (PMC3789798; doi:10.1534/g3.113.007377)
Supplement: Supporting Information [file supp_g3.113.007377_TableS1.pdf]

Table S1 Data sources for large-scale recombination and cohesin binding studies

**Publication**	**Data source**
(Gerton *et al.* 2000)	Table 1
(Borde *et al.* 2004)	Table S2
(Buhler *et al.* 2007)	Table S5 (5X background)
(Blitzblau *et al.* 2007)	Table S2
(Mancera *et al.* 2008)	Supplementary information 2: hot_spots.txt
(Pan *et al.* 2011)	Table S2 (signal intensity > 1000)
(Glynn *et al.* 2004)	Mitotic cohesin subunit (Scc1/Mcd1): Supplementary dataset 8<br>Meiotic cohesin subunit (Rec8): Supplementary dataset 9
(Kiburz *et al.* 2005)	Supp Raw Data.doc
(Kugou *et al.* 2009)	http://www.ncbi.nlm.nih.gov/geo/query/acc.cgi?acc=GSE8422
