# Supplementary material for: Global Linkage Map Connects Meiotic Centromere Function to Chromosome Size in Budding Yeast
Source: G3 (Bethesda). 2013 Oct 1;3(10):1741–51. doi: 10.1534/g3.113.007377 (PMC3789798; doi:10.1534/g3.113.007377)
Supplement: Supporting Information [file supp_g3.113.007377_SupplementaryReferences.pdf]

Supplementary references

Anscombe, F. J., 1973 Graphs in Statistical Analysis. The American Statistician **27:** 17-21.

Bishop, D. K., and D. Zickler, 2004 Early decision; meiotic crossover interference prior to stable strand exchange and synapsis. Cell **117:** 9-15.

Blitzblau, H. G., G. W. Bell, J. Rodriguez, S. P. Bell and A. Hochwagen, 2007 Mapping of meiotic single-stranded DNA reveals double-stranded-break hotspots near centromeres and telomeres. Current biology : CB **17:** 2003-2012.

Borde, V., W. Lin, E. Novikov, J. H. Petrini, M. Lichten *et al.*, 2004 Association of Mre11p with double-strand break sites during yeast meiosis. Molecular cell **13:** 389-401.

Buhler, C., V. Borde and M. Lichten, 2007 Mapping meiotic single-strand DNA reveals a new landscape of DNA double-strand breaks in Saccharomyces cerevisiae. PLoS biology **5:** e324.

Cherry, J. M., E. L. Hong, C. Amundsen, R. Balakrishnan, G. Binkley *et al.*, 2012 Saccharomyces Genome Database: the genomics resource of budding yeast. Nucleic acids research **40:** D700-705.

Gerton, J. L., J. Derisi, R. Shroff, M. Lichten, P. O. Brown *et al.*, 2000 Global mapping of meiotic recombination hotspots and coldspots in the yeast Saccharomyces cerevisiae. Proceedings of the National Academy of Sciences of the United States of America **97:** 11383-11390.

Glynn, E. F., P. C. Megee, H. G. Yu, C. Mistrot, E. Unal *et al.*, 2004 Genome-wide mapping of the cohesin complex in the yeast Saccharomyces cerevisiae. PLoS biology **2:** E259.

Jones, G. H., 1987 Chiasmata, pp. 213-244 in *Meiosis*, edited by P. B. MOENS. Academic Press, Orlando, FL.

Kaback, D. B., D. Barber, J. Mahon, J. Lamb and J. You, 1999 Chromosome size-dependent control of meiotic reciprocal recombination in Saccharomyces cerevisiae: the role of crossover interference. Genetics **152:** 1475-1486.

Kaback, D. B., V. Guacci, D. Barber and J. W. Mahon, 1992 Chromosome size-dependent control of meiotic recombination. Science **256:** 228-232.

Kiburz, B. M., D. B. Reynolds, P. C. Megee, A. L. Marston, B. H. Lee *et al.*, 2005 The core centromere and Sgo1 establish a 50-kb cohesin-protected domain around centromeres during meiosis I. Genes & development **19:** 3017-3030.

Kugou, K., T. Fukuda, S. Yamada, M. Ito, H. Sasanuma *et al.*, 2009 Rec8 guides canonical Spo11 distribution along yeast meiotic chromosomes. Mol Biol Cell **20:** 3064-3076.

Mancera, E., R. Bourgon, A. Brozzi, W. Huber and L. M. Steinmetz, 2008 High-resolution mapping of meiotic crossovers and non-crossovers in yeast. Nature **454:** 479-485.

Pan, J., M. Sasaki, R. Kniewel, H. Murakami, H. G. Blitzblau *et al.*, 2011 A hierarchical combination of factors shapes the genome-wide topography of yeast meiotic recombination initiation. Cell **144:** 719-731.

Pinsky, B. A., and S. Biggins, 2005 The spindle checkpoint: tension versus attachment. Trends in cell biology **15:** 486-493.

Stahl, F. W., H. M. Foss, L. S. Young, R. H. Borts, M. F. Abdullah *et al.*, 2004 Does crossover interference count in Saccharomyces cerevisiae? Genetics **168:** 35-48.
